# A qualitative exploration of Bahrain and Kuwait herbal medicine registration systems: policy implementation and readiness to change

**DOI:** 10.1186/s40545-019-0189-7

**Published:** 2019-10-09

**Authors:** Azhar H. Alostad, Douglas T. Steinke, Ellen I. Schafheutle

**Affiliations:** Division of Pharmacy and Optometry, School of Health Sciences, Faculty of Biology, Medicine and Health, The University of Manchester, Manchester, M13 9PT UK

**Keywords:** Herbal medicine, Medicines regulation, Drug regulatory authority, Case study, Policy implementation, Readiness to change

## Abstract

**Background:**

The Kuwaiti drug regulatory authority (DRA) lack a structured classification system for the assessment of imported herbal medicines (HMs), which leads to ambiguity in the registration process. This study aimed to examine the policy development and implementation process in an established HM registration system (Bahrain) and harness lessons to inform recommendations for a suitable HM classification system and explore implementation readiness in Kuwait.

**Methods:**

A sequential study design was chosen, with data collected in Bahrain (case 1), recommendations formed and readiness for implementation explored subsequently in Kuwait (case 2). With ethics and DRA approval in place, data sources were documentary review of regulatory policies, direct observations of HMs registration processes, and semi-structured interviews with twenty three key officials involved in the HMs registration processes. Data from all three sources were analysed thematically and findings triangulated.

**Results:**

The classification policy in Bahrain was found to be based on evidence and extensive stakeholder engagement, resulting in a clear and organised HM registration process. The availability of HMs classification policies in other DRAs, officials’ dedication and teamwork, and support by higher authority, were identified as the main facilitators in policy development and successful implementation. Barriers were the diversity of HM classifications worldwide, a lack of staff and resultant workload, and lack of training. Proposed recommendations for Kuwait were to adopt a clear definition of what constituted HMs, and to introduce a Traditional Herbal Registration based on this definition and the product’s characteristics. Interviews in Kuwait showed that almost all participants were in favour of the proposed recommendations and were in support of timely implementation. Interviewees anticipated that consistency in the HM registration process would be the main benefit, increasing reviewer’s confidence in making regulatory decisions. Interviewees also identified potential challenges which may impede successful implementation, including staff shortages, resistance to change by internal and external stakeholders, and the impact of cultural and traditional ways of working.

**Conclusions:**

Insights into the HM policy development and implementation process in Bahrain, and exploration of Kuwait’s readiness to implement resultant recommendations informed an effective implementation process for a well-designed HMs policy for Kuwait and other Arab countries.

**Electronic supplementary material:**

The online version of this article (10.1186/s40545-019-0189-7) contains supplementary material, which is available to authorized users.

## Background

Herbal medicines (HMs) have been gaining increased popularity among consumers in both developed and developing countries. According to the World Health Organisation (WHO), 60% of the world’s population, and 80% of the population in developing countries depends on HMs for their healthcare needs [1]. Global consumption of HMs has grown significantly from $20 billion in 1997 to $83 billion in 2008 [2]. Whilst a range of definitions exist for HMs, in this study, HMs are defined as “herbal preparations that are manufactured industrially in which the active ingredient(s) is/are purely and naturally original plant substance(s), which is/are not chemically altered and is/are responsible for the overall therapeutic effect of the product” [3].

The public commonly perceive HMs as safe [4], yet there are concerns about their safety too. Several adverse effects, some of them life threatening, can arise from active ingredients themselves, as well as adulteration of HMs with conventional medicines, herbal-drug interactions and inappropriate HMs formulations [5–10]. However, significant HM safety issues also arise mainly from the inappropriate regulatory classification of HMs [11, 12]. For example, in the United Stated (US), HMs are classified as dietary supplements, with requirements for evaluating quality and safety less stringent than those for medicinal products. Meaning that these products do not require assessment by the national drug regulatory authority (DRA) prior to their marketing [3, 4]. This has particular implications for many countries in the Eastern Mediterranean Region (EMR), which import the majority of their HMs from other countries including the US [13]. For a pharmaceutical manufacturing company to import and distribute HMs in these countries, it must appoint local agents, who act on behalf of the pharmaceutical company in communication with the responsible DRA to facilitate the submission of all documentation and materials for marketing the product.

Kuwait is a country that does not manufacture but imports all HMs from other countries, a HM classification system is lacking and there is no clear definition of what constitutes a HM in its DRA structure. The submission of documentation and regulatory control imposed depends mainly on how these products are classified in the country of origin [14]. This means that many HMs may escape rigorous assessment as they are marketed as dietary supplements in their country of origin. Clear classification and definition of imported HMs in the Kuwaiti DRA structure is therefore essential, in order to determine the level of regulatory control that would guide the product into the most appropriate and consistent conformity assessment for evaluating quality, safety and efficacy.

An important parameter to inform policy redesign for imported HMs in the Kuwaiti DRA structure is to explore DRAs’ approaches to HM regulations in more established systems. Therefore in 2018, a comparison of regulatory processes in five such countries was performed to investigate their existing HMs definition and classification policies. These countries were; the United Kingdom (UK), Germany, US, United Arab Emirates and Bahrain. This country comparison found a lack of consistency in the definition of what constitutes an HM, and how these are assessed and regulated. The study however recommended a universal definition for HM registration for Kuwait and other EMR countries that do not have such laws implemented [3]. The study also provided with an international HM classification option called the Traditional Herbal Registration (THR), where instead of a full registration as a conventional medicine (i.e. requiring a marketing authorisation and proven clinical efficacy), ‘plausible efficacy’ due to established history of traditional use is sufficient to assure efficacy, and evidence of bibliographic data or toxicological tests is sufficient to assure safety [3].

Nevertheless, a study of policymaking must also be concerned with an investigation of its implementation and whether implementers comply with it [15]. Therefore, relevant literature on implementation of medicines regulations including herbals was reviewed and it was recommended that more empirical work on policy implementation, driven both by relevant theory and rigorous synthesis is required [16–18]. Moreover, despite the WHO international guidelines, reports and consensus on HMs [19–25], countries still experience complications in the implementation of HM regulations, due to their diversity [26]. Analysing previous polices in similar political and cultural context can provide reliable facts and knowledge on how policies were developed and implemented, and ensures that recommendations are supported and resourced with the best available evidence and research [27]. Therefore, insights into a HM registration of an established system of a country that is similar to Kuwait is essential.

However, if proposed recommendations are to be implemented in Kuwait, some change will occur. Being ready for this change is important for successful implementation. Smith indicated that there is high risk of implementation failure if organisational or individual readiness for change is low [28]. Therefore, the best approach is an investigation of an organisation’s readiness for overall change before any implementation attempt in Kuwait; such an investigation can reveal factors about the potential success of the intended policy [28]. There are numerous studies available in the literature that describe existing frameworks to guide how readiness for change can be assessed [29–31].

The aim of this study was to examine policy development and implementation process in an established HM registration system (Bahrain) and harness lessons to inform recommendations of a suitable HM classification system in a less developed DRA (Kuwait), and explore implementation readiness there.

## Methods

### Study design

**Fig. 1:**
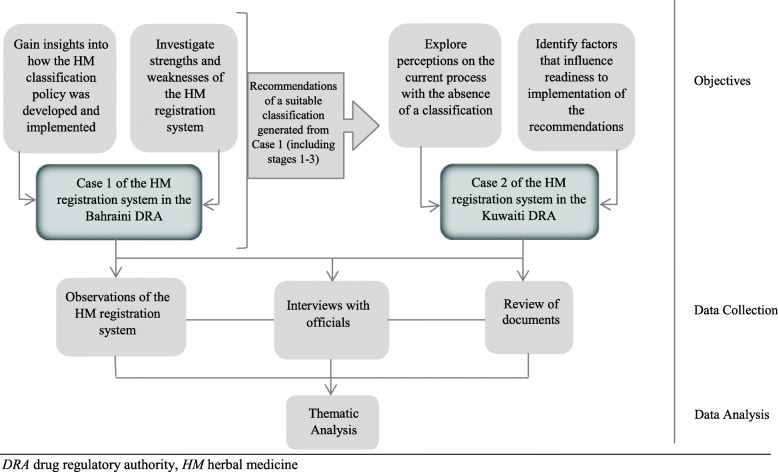
Summary of the objectives, data collection methods and data analysis carried in case 1 and case 2

The last decade of policy implementation science witnessed great interest in the use of theories and frameworks to gain insights into policy implementation and understand system strengths and weaknesses [32–34]. In this study, the conceptual model for policymaking by Anderson [15] was adopted, which consists of five steps. The first two steps have been addressed in the introduction: (1) the problem being the absence of a classification and definition for imported HM registration in the Kuwaiti DRA structure, and (2) the formulation of options for HM classifications and a definition of what constitutes a HM through a comparative study of five advance systems [3]. For steps (3) (stating content) and (4) (implementation) of the policy process, this study employed a qualitative research design in two countries (cases): case 1: an established HM system (Bahrain) and case 2: Kuwait (Fig. 1).

Case 1 focused on performing policy analysis of the HM classification policy in the Bahraini DRA using the policy triangle framework by Walt and Gilson [35], and investigated the system strengths and weaknesses to formulate recommendations for Kuwait. The DRA in Bahrain was chosen due to its geographic proximity, common culture, shared faith, and its economic and political alliance with Kuwait through the Gulf Cooperation Council (GCC). Most importantly, Bahrain is similar to Kuwait in that it imports all of its HMs from other countries through local agents. In 2016, the Bahraini DRA introduced the Pharmaceutical Product Classification (PPC) policy which clearly defines and classifies HMs [36].

Investigating the strengths and weaknesses of an established system is found useful for informing policies in unsophisticated systems [33]. Walt and Gilson explained that when researching health policy not only policy content, but also actors, context and processes need to be investigated. In this study, the four elements were used as a framework to investigate and structure data about; the policy context within which the policy was developed (i.e. context for and reasons why the policy was developed); the policy process (i.e. how the policy was developed and is being implemented); the policy content (i.e. how the content was formulated); and the actors involved (i.e. who were they and what role they played in the process). The framework has influenced the research of health policy in many countries, and has been used to analyse numerous health issues [37, 38].

Case 2 focused on identifying the current weaknesses of the system in Kuwait and readiness of staff in the Kuwaiti DRA to implement recommendations informed following case 2. Based on the Theory of Organisational Readiness for Change (TORC), Weiner [30] proposed a set of factors that the organisational members can take into consideration to formulate their change ability judgements. This study did not use the full theory process, but adopted five contextual factors from TORC which thought best achieve the aim of the study. The factors were used as a framework to structure insights into (i) policies and procedures that might influence how the recommendation could be implemented, (ii) past experiences of previously implemented policies, (iii) organisational resources that could influence readiness for implementation, (iv) organisational culture and how individuals behave towards the change, and (v) whether the change will influence the infrastructure of the organisation.

For both cases, data collection involved direct observation, documentary analysis and semi-structured interviews (Fig. 1), all undertaken by the first author who had undergone appropriate training.

#### Study participants

Approval was obtained from senior management working in the registration of HMs in Bahrain and Kuwait DRAs, who identified all senior and middle managers and all scientific reviewers who worked directly with the registration of HMs. Managers are the decision-makers of policies affecting the registration of HMs and scientific reviewers are employees who implement the HM classification policy and carry out the scientific assessment and quality control analysis for HMs. Identified participants were approached by the interviewer/observer during the visit in each authority and given a study information sheet. Managers were asked to participate in interviews, and scientific reviewers were asked to participate in observations and interviews, and if they agreed, an appointment was set.

#### Data collection

Data collection in case 1 and 2 targeted HMs in the DRAs’ departments with criteria specified in (Table 1) which are based on the current characteristics of HMs registered at the Herbal Department in the Kuwaiti DRA. Herbal teas and coffees were excluded although being one of the registered products at the Herbal Department, as these products have separate and clear definition and registration requirements as per Ministerial Decree 201/99. In Bahrain (case 1) data were collected between October–November 2017, and in Kuwait (case 2) between (April–May 2018).

**Table 1:** Data collection inclusion and exclusion criteria for HMs in Bahrain and Kuwait drug regulatory authorities

Inclusion criteria	Exclusion criteria
Herbal preparations that are manufactured industrially consist of active ingredient(s) that is/are purely and naturally original plant substance(s), is/are not chemically altered and is/are responsible for the overall therapeutic effect of the product	Other types of preparations including homeopathic products, cosmetics, medical devices and medicines containing herbal substance(s) as active substance(s) that has/have been synthesised or chemically altered Raw herbs that are not manufactured industrially HMs as teas or coffees
HMs used for curing purposes or supporting body functions	HMs that do not have a therapeutic effect or are used as flavours or additives or have a cosmetic effect
HMs for human use	HMs for animal use
HMs registration for the consumption of the general public	HMs that are not supplied for the consumption of the general public but for the purpose of supplying to specific individuals by healthcare practitioners following a one-to-one consultation
Premarketing registration of HMs (initial registration)	Post-marketing surveillance of HMs

*HMs* herbal medicines

##### Observations and documents

Data collection in each case began with non-participant observation of the HM registration process, by chronologically following the scientific reviewers’ registration process from initial request for product registration until product authorisation for marketing. The actual HMs observed remained anonymous, and it was not feasible to observe a specific product as the approval process can take months or years. Ongoing verbal consent was obtained at the start of each observation, which could involve the same or different scientific reviewers.

Detailed fieldnotes were taken during three main areas of the registration process of HMs, namely, the regulatory review processes milestones (i.e. types of activities and description of tasks), regulatory requirements and estimated timelines for key milestones of the review process. Because neither authorities have legislated timelines, in each stage, observed scientific reviewers were asked to provide the minimum and the maximum number of days it took them to complete each activity from an electronic document that records the start and end date of each activity (i.e. date of submission, date of review, date of registration, etc.). Throughout observations, the researcher asked participants some clarifying questions. Regulatory documents relating to the HM registration process such as registration requirements, registration guidelines and ministerial decrees were also analysed.

The main purpose of observations and documents review was to understand the regulatory review practices and the approaches undertaken to classify and register HMs in each authority.

##### Interviews

Face-to-face semi-structured interviews with participants at their place of work followed observations. Signed informed consent was obtained before each interview and to guarantee anonymity, a code was assigned to each participant. Interviews were audio-recorded, with permission, and for interviewees who did not want to be audio-recorded, extensive notes were taken. All interviews were conducted in English, but some responses were made in Arabic.

In case 1, interviews aimed at exploring how the Pharmaceutical Products Classification (PPC) policy in the Bahraini DRA had been formulated and implemented, the strategies and activities used, and the actors involved. Participants were also asked to reflect on their experiences on factors which might have acted as facilitators or barriers, and provide their views on the current system’s strengths and weaknesses. The interview guide was informed by a review of the policy science and implementation literature [39–43].

In case 2, interviews focused on participants’ perceptions of the current HM registration system in the Kuwaiti DRA in the absence of a classification and definition for HMs, and their perceptions and readiness for implementing proposed recommendations for a suitable definition and classification procedure for HMs. Interview questions were guided by the five contextual factors from TORC [30].

### Data analysis

All handwritten fieldnotes and audio-recordings were transcribed verbatim using Microsoft Word™ 2010. Interviews that included Arabic responses were translated into English by the interviewer who is bilingual in English and Arabic. Each collected document was reviewed and then summarised electronically describing its type, title and purpose. All three data sources were subjected to thematic framework analysis, involving five step process; familiarisation, coding, identifying a thematic framework, charting data into a matrix and interpreting the data [44].

In both cases, all data were thoroughly read for familiarity. Coding was performed by underlining segments of texts that addresses the themes in the observations and interview guide. Development of more codes was performed based on the themes in concepts and theories; for case 1 by Walt and Gilson’s policy triangle framework [35] and the strategic environment analysis to identify Strengths, Weaknesses, Opportunities and Threats (SWOT) [45] in the HM registration system, and for case 2 by Weiner’s five contextual factors of TORC [30]. Coded data from observations, documents and interviews were summarised in a matrix for each theme comprising of one row per participant or document or observation, and one column per code and inserted into corresponding cells in the matrix using Microsoft Excel™ 2010. Connections within categories were made and key similarities and differences were identified.

Ethical approval was obtained from the University of Manchester Research Ethics Committee (reference number 2017–1086-3939).

## Results

Findings are presented in two parts; each part represents a case; case 1 being for the Bahraini DRA; National Health Regulatory Authority (NHRA), case 2 from the Kuwaiti DRA; Kuwait Drug and Food Control and Administration (KFDCA). All key officials that work directly with the registration of HMs in Bahrain and Kuwait DRAs participated in the study: eight officials from the Bahraini DRA; five reviewers and three managers, and fifteen officials from the Kuwaiti DRA; nine reviewers and six managers. (for description of sources of data used in each case, see Additional file 1: Table S1). Summarised data from fieldnotes and documents about the HM registration process are illustrated to show a detailed chronological description and a map of the process in each authority. The timelines in the process are estimations, as not all reviewers keeps record of the start and end date of each activity. Quotations from interview transcripts are presented as examples of particular themes or issues. All translated Arabic quotes included in this paper are presented in a table in (Appendix 1) to show the original Arabic quote alongside the English translation. As a result of the small number of participants in each case, to maintain anonymity, the official title and positions of managers and reviewers are not mentioned.

### Case 1

#### Context, actors, content and process in the development and implementation of the PPC policy in the Bahraini DRA

**Fig. 2:**
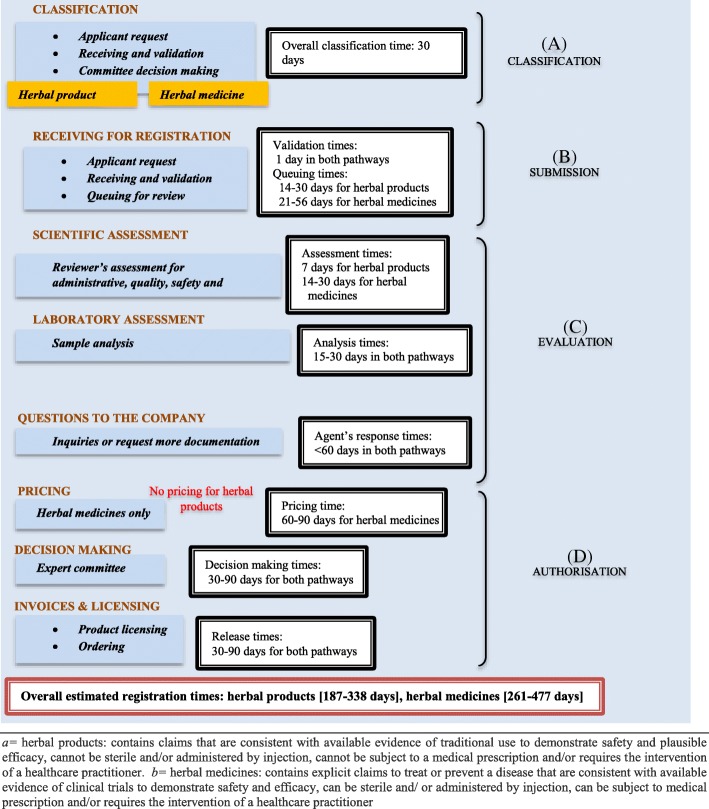
Case 1 results of the Pharmaceutical Product Classification policy development and implementation process in the Bahraini drug regulatory authority, placed within Walt and Gilson’s policy analysis triangle framework

The triangle in (Fig. 2) presents the results within Walt and Gilson’s policy analysis triangle framework. (for a clear timeline of the chronological progress of the Pharmaceutical Product Classification (PPC) policy development and implementation, see Additional file 2: Figure S1).

##### Context

Findings revealed that the development of the PPC policy was triggered by a transfer in the official body of medicines regulation in Bahrain, which coincided with the launch of the 2030 Economic Vision by the King of Bahrain in 2009. The vision aimed at developing Bahrain’s economy while focusing on improving the health sector. Emphasis was placed on the need for structural, administrative and financial independence of the regulation of the entire healthcare system by a DRA from the Ministry of Health (MOH) (Economic Vision 2030). Consequently, Law (38) of 2009 was issued by the King to transfer the regulation of health services including medicines from the MOH to the NHRA.

After the transition was complete in 2011, reviewers were facing issues in registering HMs, as at that time, many submitted HMs were reviewed as conventional medicines as per Law (18) of 1997 which was initially implemented in the MOH. The law was not clear as it did not specify explicit definitions for HMs, and participants described how agents used to submit any HM as a conventional medicine and were then unable to provide all required documents to fulfil HMs registration, which resulted in the refusal of many HMs. As one participant stated:

*“During transition registering herbals was very hard, no documents for registering herbals was there, we were not really sure what requirements we should ask companies to provide, we were confused, agents were confused” (KI6)*

The development of the PPC policy therefore started with the NHRA management developing a classification guideline to provide clear definitions for medicines and HMs.

##### Actors

Reviewed documents and interviews revealed that in 2012 the Bahraini government had invited bids to develop policies for the NHRA, following which the Dublin base consultancy International Development Ireland (IDI), was appointed. Having established the need for a classification guideline, the IDI Technical Support Services committee was established, which consisted of experts from the Irish Medical Board (IMB) and the Saudi Food and Drug Authority (SFDA) who had been chosen for their scientific and regulatory expertise in HMs and in developing regulatory guidelines. All managers and scientific reviewers responsible for the registration of pharmaceuticals and HMs in the NHRA were represented. Study participants mentioned that the main reason for including external experts in the development of the guideline was to ensure independent and guard against bias in the development process. The committee was chaired by the NHRA’s Chief of Pharmaceutical Product Regulation Department who assigned roles to the members, guided the committee in terms of tasks and process, and set a production deadline of 3 months.

##### Content

**Guideline production**

Findings from interviews revealed that the guideline was developed over a number of steps through regular committee meetings, which are described below. (for more detail and participants’ quotes, see Additional file 3: Figure S1).

The first step was a) preparing the scope. This involved the identification and analysis of previous MOH regulations with potential relevance to HMs and a comprehensive literature search. An initial scope report detailing which classification matters the guideline should discuss was drafted based on mutual recommendations from all committee members. This report recommended that an existing system that would provide international standards for HMs classification should be adapted.

The second step consisted of b) an online search of classifications in the SFDA, the IMB and authorities that are recognised by the WHO as having competent international recognised and established medicine registration systems. The NHRA investigated the European Medicines Agency (EMA), Health Canada, the UK Medicines and Healthcare products Regulatory Agency (MHRA) and the United States Food and Drug Administration (U.S FDA).

The third step was c) the formulation of recommendations. Interviews revealed that the recommendations were drafted using vote counting, and that the majority of the guideline information was *“copied and pasted”* from the SFDA, with some additional detail based on other reviewed DRAs’ classifications. When participants described that the reason for adopting the majority of the SFDA’s classifications, was that beside the political and cultural similarities between the two countries, the authority was aiming to increase harmonisation of HMs classification in the GCC countries. Such a standardised guideline was seen as facilitating a future GCC central registration system for HMs, which would allow authorisation of a single HM in all GCC member states at the same time.

In the fourth step d) the guideline was finalised in 2013 and signed by the Chief of Pharmaceutical Product Regulation Department and the NHRA Chief Executive Officer (CEO).

Participants stated that in order for the NHRA to remain independent from the MOH, it must comply with the Quality Management System (QMS) standards accredited by the International Organisation for Standardisation (ISO); an international independent organisation that offers accreditation based on evaluation of the quality, safety and efficiency of systems. According to the QMS standards, all guidelines including the PPC guideline, must be reviewed once every 4 years.

**Guideline implementation**

Participants were asked to describe the implementation process, which was defined to them as the actions taken to implement the guideline, and whether an implementation plan such as identifying required resources, training needs and projected implementation issues informed by evidence were developed. Participants confirmed that the committee did not develop an implementation plan. It became clear that the committee did not consider guideline implementation as a process that no training was provided and reviewers simply applied the guideline when registering medicines and HMs. One participant stated:

*“The implementation was not a process, the guideline was just printed and then we [reviewers] all used it to help us decide what product is a herbal and what product is a medicine” (KI1)*

However, participants revealed that shortly after the production of the guideline, the NHRA introduced the additional service of a classification inquiry which commences before the process of registration. It is available for agents who are uncertain of a product’s classification, i.e. whether it is a conventional or a HM. Following an agent’s submission of a classification application, this will be assessed by a reviewer, and a committee consisting of members from the Pharmaceutical Product Regulation Department uses factors set in the classification guideline to make its final decision.

##### Policy process

**Policy Production**

Initially the guideline had not been binding, and participants described how agents commonly failed to comply with reviewers’ classification decisions as they merely viewed them as reference. One of the participants indicated:

*“The guideline was a reference and not compulsory, sometimes we used it and sometimes we didn’t. But agents will not accept what you tell them unless it is some kind of policy” (KI5)*

Therefore, at the beginning of 2016, Decree (9) in relation to Classifying Pharmaceutical Products and Health Products came into force, making the guideline legally binging. Participants explained that the decree did not repeal Law (18) of 1997, but is to be used in combination to produce more clarity on HMs and their classification. The decree was approved and signed by the Chair of Supreme Council of Health (SCH), which is the responsible body for approving health policies in Bahrain. Members of the SCH were therefore actors in this policy process.

**Policy Implementation**

All participants confirmed that once again, no implementation plan was developed, nor was training provided to reviewers; implementation consisted of an upload of the guideline on the NHRA’s official website, which was also published in Bahrain’s official Gazette. Participants stated that management gave reviewers instructions to re-classify registered products according to the new policy while providing an adaptation period for agents to provide required documents.

**Evaluation**

When asked whether the implementation of the PPC policy was evaluated, participants explained that in order to assess the success of the policy, implementation was evaluated by calculating the total number of successful applications for HMs classified in the medicines registration, health products registration and in the classification committee prior to the legalisation of the guideline in 2015 and after. This confirmed that policy adherence was more effective after binding the guideline to a decree, resulting in a 35% increase in the registration of medicines, 33% increase in the registration of health products and 576% increase in the number of medicines and health products applications submitted to the classification committee for classification (NHRA annual report 2016).

##### Facilitators of, and barriers to, the development and implementation of the PPC policy

Having explained the development and implementation process of the policy, interviewees were asked about facilitators and/or barriers they experienced during the development and implementation process, and they identified six general themes. Participants placed much stronger emphasis on facilitators than barriers, which were themed under ‘management and collaboration’, ‘leadership’, ‘resources’, ‘nature and content of the policy’, ‘political and social influences’, and ‘staff morale and performance’ as described below. (for identified facilitators and barriers in the development and implementation phases with participants’ quotes, see Additional file 4: Tables S1, S2, S3 & S4).

**Management and collaboration**

Many participants identified the facts that the NHRA is trying to build a good reputation of their newly established regulatory body as a facilitator. They further outlined how the collaboration of the NHRA and the IDI had an important role in setting clear objectives and addressing the need to produce effective policies including a classification guideline. Moreover, many participants indicated that the cooperation and teamwork between the NHRA’s officials and the external experts from the IMB and the SFDA during committee meetings was conducive for sharing ideas and expertise.

As facilitators in the implementation phase, participants emphasised the importance of effective collaborations with other officials working in the same department, who were enthusiastic, organised and respected. Moreover, reviewers identified regular meetings with the management as important for the discussion of any products that could not be classified using the policy. Decisions that were not based on the classification policy were recorded and taken into consideration when updating the guideline. Some participants also mentioned that the management instruction to set an adaptation period for agents, which allowed the adaptation to the new system, was a strong facilitator of successful policy implementation.

In terms of barriers to implementation, all participants mentioned that the management did not provide a clear policy implementation plan to allocate adequate financial and human resources prior to implementation. According to participants, due to the urgent need in delivering the policy, assessing implementation needs were neglected.

**Leadership**

Several participants mentioned that the existence of leading figures inside the NHRA facilitated the policy development process. Specifically, participants noted the key role played by the Chief of the Pharmaceutical Product Regulation Department, when leading the production of the classification guideline committee, in terms of planning, organising and meeting the deadline for guideline completion. Moreover, participants mentioned the leading role of the CEO in approving the guideline and initiating it as a policy, as well as the continuous support and encouragement she offered them.

Strong internal leadership was also identified as an important facilitator in the implementation phase. Participants described effective leadership as having strong figures in the authority who always ensure that staff strictly follow the rules set by the authority.

**Resources**

All participants identified the availability of funding which allowed the invitation of external experts with the appropriate skillsets as an important facilitator in the development phase. Additionally, as the NHRA deciding to adopt an existing classification system, the online availability of and access to data on HMs classifications and other HMs regulations and guidelines were perceived as important resources, which facilitated the production of the policy. Moreover, participants indicated that the participation of reviewers in the production of the policy facilitated a rapid implementation, as reviewers had a good understanding on how to implement the policy in practice and therefore no training was required.

Regarding barriers, many participants identified the lack in experience and knowledge in HMs, and the lack in decision-making techniques by NHRA members due to lack in training.

**Nature and content of the policy**

In the implementation phase, all participants indicated that the nature of the policy itself was a strong facilitator of implementation as it contained clear guidance that directs officials and agents on how products should be classified. Participants explained that the policy saved them time, effort and made their job easier. Moreover, some participants mentioned that the facts that the policy had been based on classifications in countries that Bahrain imports from was a key facilitator, as it meant that these products experienced a smoother classification process because of their similarity in regulatory statuses.

Many participants perceived the lack of a universal classification for HMs and the diversity of worldwide herbal regulations as one important barriers in the development phase, creating difficulties for the committee when deciding which classifications to adopt.

As in the development phase, the diversity of classification of HMs worldwide and the continuous change in HMs regulations were also found as challenges in the implementation phase. Participants stated that it was therefore difficult to implement the policy effectively, even when updating the guideline regularly, as complete compliance with the guideline still remained difficult.

**Political and social influences**

Participants identified the usefulness of previous and current national and international policies in making the case for development of a clear classification policy in Bahrain as clear facilitator in the development phase. For instance, Law (18) of 1997 for the registration of HMs was found to be incoherent and controversial, and was used as a justification to demand action. Additionally, some participants mentioned that Bahrain being a member of the GCC facilitated the production of the policy by adopting another member’s classification; Saudi Arabia, and introduced the idea of the policy itself through communications with other members during the GCC central registration meetings. Moreover, many participants identified that gaining structural independence of the NHRA from the MOH was the most important factor that facilitated the production of the classification policy since the approval of policies do not have to go through the lengthy MOH policy process anymore. Finally, strongly linked to leadership, some participants indicated that the existence and the support of the SCH as a across-sectoral organisation responsible for approving all policies produced by the NHRA in an efficient and quick manner was viewed as a significant facilitator to policy development.

As facilitators in the implementation phase, the change in the political climate and the autonomy of the NHRA and its independence from the MOH was once more mentioned by most of the participants, and was seen as an effective factor facilitating the implementation of the policy. Participants explained that after the transition of the regulatory services from the MOH to the NHRA, the MOH could no longer influence the decision-making of applications for products registration. Additionally, many participants identified the binding of the guideline to a decree as facilitating implementation. They explained that the decree illustrates that any violations to its provisions are subject to sanctions outlined in the Law No. (18) of 1997, and therefore violators would be guilty of an offence punishable by imprisonment and a payment of a fine. For this reason, agents were found more adherent to the new classification system.

In terms of barriers in the implementation phase, some participants mentioned that they experienced resistance from some agents to comply with the new classifications, particularly before it became legally binding.

**Staff morale and performance**

As facilitators in the development phase, many participants mentioned that the efficiency of committee officials who participated in the development of the policy and their consistency and commitment in finalising the guideline was a strong facilitator that led to the delivery of the guideline on time.

As facilitators in the implementation phase, similar to the development phase, many participants mentioned staff motivation and dedication as an important facilitator. Participants explained that their motivation in implementing the policy effectively came from them valuing the influence of the regulatory authority in protecting the consumer. They outlined that they have a responsibility to protect the public by complying with the policies of the authority, as they expressed their concern regarding the Arabic culture and the low awareness of consumers using HMs *“like sweets”*, therefore participants believed that by implementing the policy effectively this would safeguard the public.

#### The registration process of HMs in the Bahraini DRA

Having discussed the development and implementation processes of the PPC policy, it was essential to investigate how HMs are currently registered and classified in the NHRA after the implementation of the policy. Findings from observations revealed that a HM in the NHRA is classified under two registration pathways, either as a herbal product under the Health Products Registration Department (simplified registration) or as a herbal medicine under the Medicines Registration Department (stringent registration). Table 2 defines HM within each pathway.

**Table 2:** Herbal product and herbal medicine definitions at the Bahraini drug regulatory authority (Pharmaceutical Product Classification guideline)

➢ Herbal product “health product containing as active substances, herbal substances or herbal preparations, alone or in combination. It should not carry medicinal indications or make medical claims that are unsuitable for self-diagnosis and self-treatment i.e. without the intervention of a licensed healthcare professional. Any claims made in association with herbal products should be consistent with available evidence regarding the safety and traditional use of those products. A herbal product cannot be sterile, be administered by injection, be subject to a medical prescription, necessitate the intervention of a licensed healthcare professional”	
➢ Herbal medicine “any substance or combination of substances presented as having properties for treating or preventing disease in human beings; or any substance or combination of substances which may be used in or administered to human beings either with a view to restoring, correcting or modifying physiological functions by exerting a pharmacological, immunological or metabolic action, or to making a medical diagnosis”	
In both definitions:	
➢ Herbal substances are referred to as “whole, fragmented or cut plants, plant parts, algae, fungi, lichen in an unprocessed, usually dried form but sometimes fresh. Certain exudates that have not been subjected to a specific treatment are also considered to be herbal substances”	
➢ Herbal preparation is “obtained by subjecting herbal substances to treatments such as extraction, distillation, expression, fractionation, purification, concentration or fermentation. These include comminuted or powdered herbal substances, tinctures, extracts, essential oils, expressed juices and processed exudates”	

**Fig. 3:**
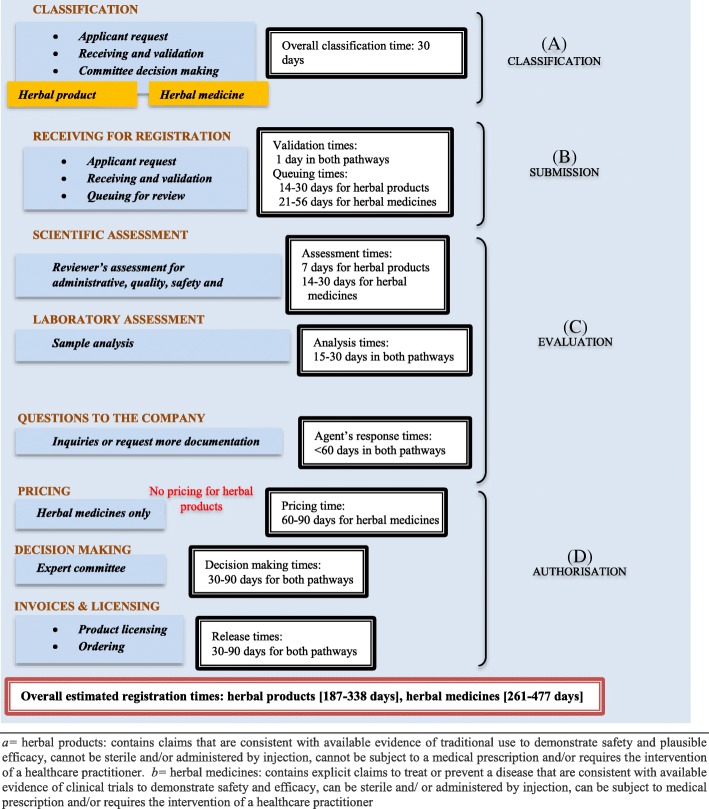
Process map of herbal products^a^ and herbal medicines^b^ registration with estimated times in milestones, extracted from fieldnotes recorded during observations at the Health Products Registration Department and the Medicines Registration Department in the Bahraini drug regulatory authority

From the analysis of fieldnotes and collected estimated timelines of the registration process (Fig. 3) it was revealed that overall, the regulatory process for herbal medicines is more rigorous and therefore takes longer to register than herbal products. For establishing a common ground for comparing the HM regulatory review in both pathways, the processes were divided into A) classification, B) submission, C) evaluation and D) authorisation (for description of the regulatory process including similarities and differences in the process between the two pathways, see Additional file 5).

#### SWOT analysis for the HM registration system in the Bahraini DRA

A SWOT analysis was conducted based on interview responses (for participants’ quotes with identified SWOT, See Additional file 6: Table S1).

The most commonly identified strengths were the motivation of the NHRA to continuously improve and amend the system, the clarity and transparency of the review procedure which established set of rules, and the existence of a scientific committee which makes decisions whereby hidden biases are overcome and inappropriate decisions limited. Some participants also identified the availability of clear guidelines and Standard Operating Procedures (SOPs) that assist the pharmaceutical industry and professionals in their compliance as a strength. The use of an electronic system for the review procedure was seen as a further strength, as this limits making errors, misplacing files and missing data.

The most commonly identified weaknesses were the lack in organisational structure and hierarchy resulting in poor communication between departments, poor management of financial resources between departments causing some departments to benefit from the training opportunities more than others, and extreme lack in human resources resulting in heavy workloads.

The opportunities identified by the largest number of participants was the independence of the authority from the MOH, which gives the NHRA absolute power in providing advanced regulatory practices without interference of external interests. The NHRA’s ability to expand and improve their regulatory services through knowledge transfer and sharing of best practices from the GCC Central Drug Registration meetings and collaborations with other international agencies was seen as a further important opportunity.

The threats mostly identified were the growing trade of counterfeit HMs worldwide, and the absence of a pharmacovigilance system in the NHRA to monitor adverse drug reactions. Some participants also described the threat of importation from countries with weak HMs regulations, and consumers obtaining unsafe HMs through the internet, where products are neither inspected nor assessed locally.

### Case 2

#### The registration process of HMs in the Kuwaiti DRA

**Fig. 4:**
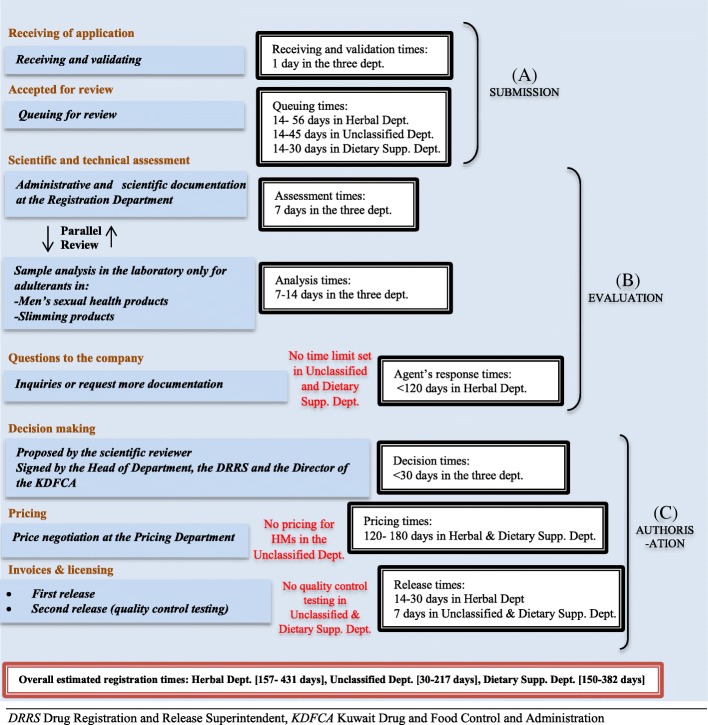
Process map of HMs with estimated times in milestones, extracted from fieldnotes recorded during observations at the Herbal Department, Unclassified Department and Dietary Supplement Department in the Kuwaiti drug regulatory authority

Following the detailed analysis of policy development, content and implementation in Bahrain, informed recommendation for an improved HM regulatory system. These recommendations were taken to Kuwait, case 2, where views on these recommendation were sought and the authority’s readiness for change was also explored. Before this, participants were asked to describe the current system of HM regulation/ registration, and observations revealed that under the current system in the KDFCA, HMs can be allocated and reviewed in three departments; Herbal Department, Dietary Supplement Department and Unclassified Department (Fig. 4). To facilitate effective comparison between the HM regulatory review in the three departments, the processes were divided into three phases, namely, A) submission, B) evaluation and C) authorisation. Overall, analysis showed that the regulatory process for HMs in the Herbal Department was more rigorous and therefore took longer than in either the Dietary Supplement or Unclassified Departments. Unlike products at the Herbal and Dietary Supplement Departments that must undergo specific pricing procedure by the KDFCA, products registered at the Unclassified Department do not get priced by the KDFCA, but are priced according to the company’s desires. (for full description of the regulatory process including similarities and differences in the HM process in the three departments, see Additional file 7).

#### Perceptions on the current system in the absence of a classification

All interviewees expressed concerns over the absence of a clear definition and classification procedure for HMs (Table 3). Many participants described that there was confusion on how to carry out the regulatory process despite the existence of the Ministerial Decrees, and that clear and decisive regulations were required so that incorrect and inconsistent decisions could be avoided. Moreover, many participants described their difficulties in deciding where to register products which included herbal and other ingredients such as vitamins and minerals. They explained that they did not have sufficient information to guide them nor were they able to use the regulations to *“back them up”* when agents and pharmaceutical companies argued to register products in certain departments. Moreover, other participants explained that due to the lack of clarity on what constituted a HM lead agents to register products according to their registration status in the country of origin.

**Table 3:** Perceived issues resulting from the absence of a clear definition and classification for HMs, with participants’ quotes extracted from transcripts of interviews with officials at the Kuwaiti drug regulatory authority

Identified issues	Participants’ quotes
Confusion on how to carry out the regulatory process	*“We are not sure that some products should be classified in the unclassified unit. So you see here there is a confusion. Reviewers are confused to where best classify the product because there is no clear guideline to follow. Sometime I am lost” (KI13)*
Difficulties in deciding where to register products that include a mixture of herbs and other ingredients	*“We receive products that contain many herbs and many vitamins, it is hard to make a conclusion whether this product is a herbal product or a vitamin product. What is happening that we are puzzled. How should we classify herbal products with vitamins? According to what? Is it according to the number of herbs? What if we receive a product that has a number of herbs are equal to the number of vitamins? And what about the pure herbs? “(K12)*
Participants feel that agents have more power	*“…the sad thing that the power of agents and companies exceeds our power. We are the supervisory authority, we should have the power” (KI17)*
Restrictions as a result of the term herbal medicine	*“The terms are restricting us. We do not have something called herbal supplement. When the agent hears the name medicine, they are frightened because this means stricter registration process so they go to the unclassified unit to register this herbal supplement” (KI22)*
Inappropriate pricing system	*“I do not get the concept of regulators in not forcing all products to be priced. From their point of view, they claim that dietary supplements are not a mandate; you do not take it to increase your life expectancy or to treat or cure a disease you have… people are taking them as a luxury. But if this is the case, then why do you price products in the herbal department? Not all of them are medicinal and not all of them have a medicinal use. My opinion everything must be priced” (KI16)*
The lenient regulatory process in the Unclassified Department	*“As a Herbal Department, we should have all herbal products that have purely herbal active ingredients registered here. The unclassified unit is here to provide a gap for agents instead of waiting a long time for their products to be registered at the herbal unit…” (KI10)*
Inconsistency and duplication in registration of many HMs with the same active ingredients and characteristics	*“What happens now is that the agent submits the product to the Unclassified Department while the product must be registered in the Food Department. And you find another agent, submits this exact active ingredient of a product in the food supplement and they both get registered but in different departments. But tell me, which one is the right department? How could we know for sure if we do not have clear regulation or information indicating what circumstances makes an herbal product a herbal medicine, food supplement or dietary supplement or even a vitamin” (KI16)*

*HMs* Herbal medicines

Because of lenient regulatory process in the Unclassified and Dietary Supplement Departments, where many HMs are marketed without testing, many participants considered the scientific analysis of products prior to market release as the most important requirement in order to maintain product quality and safety. In fact, many participants stated that all products should be classified and that the Unclassified Department should be removed. Some participants were against the MOH not pricing products in the Unclassified Department, as this meant that prices of unclassified products were very high, which they considered to be *“unjustifiable”* and that is *“not fair”* to consumers and patients.

A further problem that was identified by many participants was that different HMs with the same active ingredients and characteristics can be registered in more than one department, causing inconsistency and duplication in product registration. This led companies to complain about unfair and uncompetitive disadvantage.

#### Perceptions on implementing proposed recommendations

Having discussed views regarding the absence of a clear definition and classification for HMs, participants were asked about their opinions on implementing the proposed policy recommendations for HM definition and classification (Appendix 2), which had been given to them prior to the interview. These proposed recommendations aimed to promote harmonisation of HMs regulations, and were based on the findings from the five-country comparison [3] and the Bahraini case study 1. The recommendations consisted of 1) adopting a universal harmonised definition of what constitute a HM for the purpose of registration and specifying a directive that state that all herbal preparations matching the proposed definition must be assessed under one department, the Herbal Department, 2) under the Herbal Department, HMs should be registered under one of the two registration pathways a) Traditional Herbal Registration (THR) (simplified pathway) and b) Herbal Medicine Registration (HMR) (standard pathway), and 3) the decision for classifying a HM under the either pathways depend on the ability to prove the product’s efficacy (i.e. in THR ‘plausible efficacy’ due to established history of traditional use is sufficient to assure efficacy, whereas, HMR requires full registration similar to a conventional medicine registration requiring a marketing authorisation and proven clinical efficacy).

Overall, there was real enthusiasm about introducing a HM classification policy by almost all interviewed participants and they described it as an *“urgent need”.* Some of the expressions were:

*“This is an excellent idea. This will solve many problems. You are not proposing that these products are registered straight away, this only means that following the guideline we classify the products in the right department” (KI16) “We need it [classification policy]. I totally agree” (KI15) “…from the beginning of the registration, it is much, much better that a product be classified” (KI20)*

However one participant believed that a classification policy was not needed in Kuwait, due to restrictions this would impose. This participant felt that reviewers were experienced enough to make the right decision, and that Kuwait, as an importing country, should be align with the product status in the country of origin. The remaining participants described the benefits (Table 4) as saving time for both reviewers and agents by allocating the HM to the right department at the start, consistency in the HM registration process, increasing reviewer’s confidence in making decisions and improving consumer’s safety by assuring that all HMs are assessed correctly before marketing.

**Table 4:** Perceived benefits for implementing the proposed recommendations of a HM definition and classification, with participants’ quotes extracted from transcripts of interviews with officials at the Kuwaiti drug regulatory authority

Benefits	Participants’ quotes
Saving time for both reviewers and agents	*“…it will help and solve a lot of problems, and will assure us and the agents, that the product is registered under the correct registration department with the correct requirements from the beginning of registration,* and *products will not be transferred from one department to another in order to make a decision.” (KI12)*
Consistency and clearness in the HM registration process	*“I think this [classification policy] will make things easier and clearer. Today, to be honest, there is confusion and disorder in classifying products. Look, to be honest, during one year, many products were transferred, and most of them because of the complaints of big companies against their competitors. And if it wasn’t them, nothing will be changed. So this [classification policy] will change many things and will put things into order” (KI10)*
Increasing reviewer’s confidence in making decisions	*“I noticed something else that agents, once you tell them that this is a policy or this is a guideline… well, this is out of my hands, they actually adhere with you. But if there is no policy or no guideline, even if you told them many times, well, this shouldn’t be registered here, they won’t listen to you because there is no proper guideline they have to follow” (KI14)*
Improving consumer’s safety	*“Our requirements for herbals [in the Herbal Department] are excellent very strong requirements, almost similar to the pharmaceutical registration requirements so if all HMs are registered here this will make sure that side effects are less to appear and this will increase the safety of consumers* “*(KI11)*

*HMs* herbal medicines

##### Contextual factors of the readiness for implementing the proposed recommendations

Participants were asked to provide their views on the five contextual factors informed by TORC that would affect the authority’s readiness to implement the proposed recommendations; a) policies and procedures, b) past experience, c) organisational resources, d) organisational culture and e) organisational structure. (for identified sub factors and participants’ quotes, see Additional file 8: Table S1).

**Policies and procedures**

Many reviewers stated that the authority’s management lacked motivation to introduce new policies, because employees are promoted as a result of the number of years they have worked for the authority rather than the quality of their work and ideas they proposed. Some interviewed reviewers suggested that incentives may be helpful, and that management may benefit from the inclusion of different, younger people who may be more motivated to introduce new ideas that suite the new era. Indeed new staff had recently been recruited for management positions, and interviewees were optimistic that the system would improve as a result of this.

Views between interviewed reviewers and management differed regarding the level of their involvement with each other when developing policies. Most reviewers considered their involvement in policy development as important. Yet reviewers feel that their views were not encouraged by management who did not value their specialised opinions. Management, however confirmed that they would include whoever was necessary in the policy development process.

Decision-makers were also asked to share their views regarding the process of approving a HM classification policy. They viewed updating the current Ministerial Decree as the best approach; they also agreed that the power to impose penalties would give the authority the power over agencies and companies who were non- compliant. Many participants agreed that parliament should consider separating the KDFCA from the MOH to become a fully independent authority and give the KDFCA the autonomy to improve and implement regulations without the need for the lengthy process approval.

**Past experience**

Participants were asked about issues that, based on their past experience, would need to be considered before policy implementation. Most participants emphasised the authority looking into the different regulations in different countries and prepare a policy that was compatible with international regulations. Other participants added that the authority must consider the exporting countries’ classifications. Many participants also stated that it would be essential to have continuous discussions and regular meetings with the employees who would be implementing the policy to discuss any issues they encountered. Participants also stressed the importance of regularly reviewing the policy according to international policies to ensure it remained up-to-date.

All participants recognised that the introduction of the classification would result in agents disagreeing with the decisions made, which they would try to change. Participants therefore suggested an implementation period for agents to get used to the requirements. Some participants also recommended that there should be a formal right to appeal on decisions made, as this would give agents a level of advocacy. Publication of the policy on the authority’s official website was seen as further increasing both transparency and compliance.

**Organisational resources**

Once the HM classification was in place, many products previously registered under the Unclassified or the Dietary Supplement Departments would need to be re-classified. Therefore, many participants considered it to be essential to increase the number of reviewers, particularly as the authority already faced significant staff shortages. Other participants stated that it would be vital to also have herbal specialists who understand the science behind herbals and are able to solve confusion which may occur.

All participants noted that reviewers would require training, including on international guidelines and regulations in HMs from other countries. All participants recognised that the implementation would require financial resources to ensure enough staff, training courses and technical methods. Some participants did not consider this a strain, because the MOH held a significant amount of financial resources.

**Organisational culture**

All participants described what is known as mediation or favouritism; giving preferential treatment to one person at the expense of another, are cultural norms which also impact regulatory decision- making. Many participants recognised that in order to overcome these cultural challenges, it would be important to appoint people with high integrity and honesty. Other participants suggested that one way of dealing with the risk of mediation and favouritism was to appoint more than one reviewer to decide on the classification of a HM.

Moreover, many participants described that the current system whereby agents simply turning up without appointments was not conducive to an independent and organised workflow. Having to respond to agents at any time caused reviewers stress and meant there were no clear boundaries between agents and reviewers. They recommended that there should be a separate office or reception that welcomes agents, deals with their requests, and organises appointments for receiving files.

**Organisational structure**

Some participants thought that the implementation would require organisational restructure, others did not. Those who advocated a new structure suggested that it would be essential to introduce the policy with a separate classification department that is only responsible for classifying products.

## Discussion

This study used Anderson’s conceptual model for policymaking [15] to analyse the Pharmaceutical Product Classification (PPC) policy in Bahrain, including the system’s strengths and weaknesses, which informed recommendations of a suitable HM classification procedure for Kuwait. These recommendations were then used to explore Kuwait’s readiness towards implementing. Each of Anderson’s steps applied a policy model to guide study design and frame analysis, which delivered a valuable and novel procedure for analysis and interpretation.

Case 1 provided insights in the policymaking process of the PPC policy in the Bahraini DRA and showed that contextual factors were important catalysts to setting the NHRA’s agenda in improving their policies, particularly the separation of the authority from the MOH and a desire to establish an internationally recognised robust system. The importance of the involvement of international experts in the policy process was also revealed, which played a major role in agenda setting and adoption of a policy which outlines criteria for classification decisions and solve HMs registration issues. In combination with a five country comparison [3], the findings from case 1 informed recommendations for a suitable definition and classification procedure for Kuwait which is similar to the European Directive on Traditional Herbal Medicinal Products and the Bahraini PPC policy [3]. Specifically, the recommendations were to adopt a harmonised definition of what constituted HMs, and to introduce a Traditional Herbal Registration, to ensure that the efficacy of traditional herbal medicinal products is considered plausible without the need for conducting extensive clinical studies. These recommendations were used in case 2 to investigate the Kuwaiti authority’s readiness for implementation, which revealed positive responses and high motivation from officials.

Both the logic and research evidence in policy implementation and readiness for change have concluded that there is a high chance of implementation success if the members’ willingness to adapt to the change is high [31, 46]. However, other features also have a great influence on the success or failure of policy implementation [17, 18, 47, 48]. Using perspectives in literature and insights into the two investigated cases, five common features were identified which the Kuwaiti DRA must consider. These features are: management support and leadership, employees’ involvement in the policymaking process, organisation cultural context, implementation planning and allocation of resources, and the organisation’s autonomy.

Leadership in management is important in providing commitment, motivation and direction to employees [49, 50]. From case 1, the Bahraini DRA had leading management figures who engaged with reviewers and motivated them throughout the policy change process. In case 2 however, reviewers raised a lack in communication and appreciation of management, which ought to change in Kuwaiti DRA management, so that leadership can inspire employees and engage them in the change initiatives.

Involving employees in the policymaking process aligns with Hajar and Weagenaar, who note that policymaking has to become more interpretive (less top down), involving people’s stories, views and beliefs [51]. In case 1, the Bahraini DRA involved all reviewers in the development of the policymaking processes, making it easier for reviewers to understand and implement the policy in practice. In case 2, reviewers explained that they currently have limited opportunity to interact with management, but interviews with management indicated that they would involve relevant reviewers in the development of the policy.

Pharmaceutical industry gain significant profits following successful registration and pricing of their products, and their interests have been perceived to influence the policy implementation significantly [38]. In case 2, resistance of agents and pharmaceutical companies to changes and the impact of cultural and traditional ways of working was affecting some important regulatory decisions in the Kuwaiti DRA. In case 1 however, Bahrain DRA’s decisions were based on a clear system and transparent regulatory procedure with the final decisions performed collectively through a committee which made it difficult for agents and pharmaceutical companies to modify or influence regulatory decisions. These features could be adopted by the Kuwaiti DRA to prevent the possibilities of conflicts of interest and/or the culture of favouritism and corruption entering the system. Moreover, to increase compliance, similarly to the Bahraini DRA, the Kuwaiti DRA should consider binding the guideline to a decree, so that employees and agents are legally obligated to comply with the content of the guideline.

Before any attempt for implementation is made, it is important that resources for potential and projected implementation needs are identified and anticipated [31]. In Case 1, policy reflected the “quick-fix” mentality of policy-makers [52] which meant setting an implementation plan was neglected. This resulted in implementation challenges, such as lack of expertise in HMs, lack of regular training, and workload due to limitation in staff. Case 2 indicated that there was potential for similar challenges upon implementation which would need to be considered. Identifying appropriate monitoring and evaluation measures for implementation including allocation of evaluation responsibilities and monitoring resources, also need to be addressed in the planning phase [53]. In case 1, although the Bahraini DRA conducted evaluation of the PPC policy by calculating the number of successful products applications prior and following implementation of the policy, the evaluation did not specify the number of successful classifications for HMs alone, but included all product types without specifying the number of each type. Other critical evaluation aspects that the Bahraini and the Kuwaiti DRAs should consider include obtaining reviewers’ and local agents’ feedback (e.g. through questionnaires, complaints, meetings or workshops), and undertaking inspections to monitor classification consistency, accuracy and compliance through observing reviewers’ performance and tracking of applications [54].

Case 2 exemplified that one of the main deficiencies in policies is because the Kuwaiti DRA is structurally, administratively and financially under the autonomy of the MOH, slowing down policy development and implementation. However, Kuwait’s regulatory authority works independently from all the other divisions and departments within the MOH, which leads to it important role not being sufficiently recognised by the government. This makes it very difficult for the regulators to persuade the MOH to improve and approve the policies within the Kuwaiti DRA [55]. In case 1, the separation of medicines regulation from the MOH provided the Bahraini DRA the autonomy to produce regulations and approve them without the interference and the lengthy process of approving them through the MOH. It is therefore recommended that the Kuwaiti government considers separating the Kuwaiti DRA from the MOH to become a fully independent authority.

This study has several strengths. In both cases, the investigation of the regulatory processes triangulated the findings from three different sources, namely documents, direct observations and in-depth interviews to provide an accurate picture of the regulatory processes and staff experiences in each regulatory authority. Moreover, there was a high participation rate, with all key officials involved in the HM registration process in both authorities participating.

The study has a number of limitations. First, in case 1 recall bias could be an issue, as participants had to retrospectively reflect on the policymaking and implementation process of the PPC. However, recall bias was counteracted by validating findings by document review. Second, in both cases it was not feasible to observe individual products, and in both cases, timelines were estimated but not validated. Finally, as both cases only targeted participants who work directly with HMs, the views of other stakeholders such as agents and consumers were not explored.

## Conclusions

Increasing consumer demand for HMs, and possible undesirable effects resulting from the consumption of HMs, necessitated that national DRAs sensibly update their HM policies to safeguard the public. This study makes a unique and novel contribution to the HM policymaking literature by generating insights from one of the DRAs (case 1: Bahrain) which had recently updated their HM registration system. Using Anderson’s policymaking steps, a detailed analysis of policy development, content and implementation in the Bahraini DRA (case1), together with a previous document analysis that investigated HMs laws in advanced systems, provided evidence-based lessons for effective HMs regulation. The recommendations included a clear definition of what constitute HMs, and an introduction of a Traditional Herbal Registration based on this definition and the product’s characteristics. Subsequently, these recommendations were examined for implementation readiness in an unsophisticated HM system in Kuwait (case 2), concluding that the potential implementers’ readiness for implementation was high.

It is anticipated that lessons from both case studies can help guide other countries with improving their HMs policies. Study methodology can be adopted in future policy case studies including comparative case studies. Future research could incorporate the views and perceptions of other stakeholders such as HMs users, agents who register HMs and manufacturers/ industry.

### Additional files

Additional file 1: Description of data sources used in the illustration of findings in case 1 and case 2 (DOCX 14 kb)

Additional file 2: An analysis of the Context, Actors, Content and Process in the development and implementation of the Pharmaceutical Product Classification policy in the Bahraini drug regulatory authority (DOCX 58 kb)

Additional file 3: An analysis of the production stages of the Pharmaceutical Product Classification guideline at the Bahraini drug regulatory authority (DOCX 31 kb)

Additional file 4: An analysis of facilitators and barriers in the development and implementation stages of the Pharmaceutical Product Classification policy in the Bahraini drug regulatory authority (DOCX 28 kb)

Additional file 5: An analysis of the classification and registration process of herbal products and herbal medicines at the Bahraini drug regulatory authority, including similarities and differences between the two pathways (DOCX 21 kb)

Additional file 6: An analysis of Strengths, Weaknesses, Opportunities and Threats of the current HMs registration system at the Bahraini drug regulatory authority (DOCX 19 kb)

Additional file 7: An analysis of the registration process of HMs at the Herbal Department, Dietary Supplement Department and Unclassified Department in the Kuwaiti drug regulatory authority, including similarities and differences between the three departments (DOCX 19 kb)

Additional file 8: An analysis of factors affecting the Kuwaiti drug regulatory authority’s readiness to implement the proposed recommendations (DOCX 21 kb)

## Data Availability

The datasets used or analysed during the current study are available from the corresponding author on reasonable request.

## Appendix 1

**Table 5:** Translated Arabic quotes from interviews used in the study

Arabic Quote	English Translation
*“لأننا نحاول ناخذ شهادة الآيزو، وعشان ناخذها، كل المستندات، و الأوراق الإرشادية ضروري تتجدد كل أربع سنوات. حتى لو ماكان في ضرورة للتغير، على الأقل التاريخ بالمستند او الأوراق لازم يتجدد” (KI8)*	*“As we are trying to achieve the ISO certification, to achieve this certificate, all documentations, guidelines must be revised every four years. If there are no amendments needed, at least the date of the version must be updated” (KI8)*
*“عشان نوفر الوقت و الجهد، قلنا نتبنى قوانين و إرشادات دولة نثق فيها، ونتبنى تصنيفها للأعشاب. ليش لازم نسوي عمل مكرر ؟ المفروض مانسوّي أي شي من الصفر، خلينا نجرب نغيّر شغلات يلّي توالم دولتنا و نتبنّى الباجي” (KI8)*	*“To save time and effort, we said let’s adopt the policies and guidelines of a country that we trust and we adopt their herbs classifications. Why do we have to do a duplicate work? We should not do something from scratch, just try and amend things that suit our country and adopt the rest” (KI8)*
*“الشيء المحزن إنّه قوة الشركات و الوكلاء تضاهي قوتنا، إحنا السلطة المشرفة، احنا إلّي مفروض تكون عندنا القوة” (KI17)*	*“The sad thing that the power of agents and companies exceeds our power. We are the supervisory authority, we should have the power” (KI17)*
*“متطلباتنا بالأعشاب [في قسم الأعشاب] ممتازة، متطلبات قوية جداً، تقريبا تشابهه متطلبات تسجيل الأدوية، فإذا كل أدوية الأعشاب تتسجل هنا، هالشيء راح يأكد إنّه الأثار الجانبية راح تكون اقل ظهور وهذا يزيد من سلامة المستهلكين” (KI11)*	*“Our requirements for herbals [in the Herbal Department] are excellent very strong requirements, almost similar to the pharmaceutical registration requirements so if all HMs are registered here this will make sure that side effects are less to appear and this will increase the safety of consumers* “*(KI11)*
*“خبرتنا بالكيمياء والأدوية العادية أكبر من الأعشاب. تأسيس تصنيف أو حتى قوانين تضبط الأعشاب بغاية الصعوبة؛ الموضوع مو بس أسود و أبيض، الموضوع معقّد و إحنا ماعندنا الخبرة. للأسف، ماتدربنا على أسس إتخاذ القرارات. كل شي إعتمد على اجتهادنا الشخصي وقراءتنا و سؤالنا خبراء ثانين” (KI3)*	*“Our experience in Chemistry and conventional medicines is more than herbals. Establishing classification or even policies to regulate herbs is extremely difficult; the topic is not just black and white, it is a complicated subject and we don’t have the expertise. Unfortunately, we didn’t receive training on decision-making techniques. It all depended on our personal effort and reading and asking other experts” (KI3)*
*“هذي طبيعة عملنا عرفتي، إحنا مانستلم مكافآت وعلاوات لما ننجح بتطوير السيستم، او لما نشتغل من جد، احنا نترقى من سنوات الخبرة، فالموظفين مو متحفزين يعدلون بالسيستم، ماراح يستفيدون من هالشي” (KI11)*	*“It is the nature of our work you know, we do not receive rewards and bonuses when we succeed in improving the system or when we work really hard, we get promoted because of the years we serve, so the staff is not motivated to improve the system, they will gain nothing from that”(KI11)*
*“تجديد الدم، يحسّن السيستم” (KI10)*	*“Renewing the blood, improves systems”(KI10)*
*“شوفي، مشكلتنا الوحيدة اهي مع قسم الأنكلاسيفايد، مو واضحين. هالقسم مو منطقي” (KI16)*	*“Look, our only problem is with the unclassified department. They are not clear. This department is not logical” (KI16)*
*“الواسطات والمصالح عمرها ماتمشي بمجوعة، تمشي بس بين شخصين أثنين، فشنو نقدر نسوي، انه اكثر من مقيّم يقرر تصنيف المستحضر، مو بس واحد” (KI12)*	*“Mediation and making favours, can never work on a group, it works individually secretively between two people, so what we can do is that more than one reviewer decides the classification of the product not only one” (KI12)*

*HMs* Herbal Medicines, *ISO* International Organisation for Standardisation

## Appendix 2

### Proposed policy recommendations of a definition and a classification procedure for the registration of imported HMs in the Kuwaiti drug regulatory authority

1. **Definition:** A reasonable anticipated step would be the possibility for Kuwait to adopt a universal harmonised definition of what constitute a herbal medicine for the purpose of registration that would guide the product into the most appropriate conformity assessment. A proposed definition could be:
  *“Herbal preparations made from one or more herbs as the active ingredients, which may additionally contain excipients, however finished products to which the active substances has been chemically altered or added, including synthetic compounds and/or isolated constituents from herbal material, are not considered herbal. Herbal preparations are intended for prophylactic, therapeutic, or other human health benefits”* (WHO, 2000).

In addition to adopting a universal definition for HMs, in order to prevent HMs from being categorised as dietary supplements, which as a result circumvent detailed assessment procedures, a directive should also be specified that **all herbal preparations that match the proposed definition must be assessed under the Herbal Department.**

2. **Registration pathways:** It is proposed that under the above definition, HMs could be divided into two registration pathways; a) Traditional Herbal Registration (THR) (simplified pathway) and b) Herbal Medicine Registration (HMR) (standard pathway). The THR is to create a simplified registration procedure for all traditional HMs not fulfilling the requirements for the HMR pathway. The main registration requirements for the THR and HMR would therefore be as follows:

**Table:**

Main registration requirements	THR (simplified registration)	HMR (standard registration)
Evidence of quality	GMP standards and QC tests	GMP standards and QC tests
Evidence of safety	Evidence of safe traditional use from published scientific literature or international monographs	Toxicological studies
Evidence of efficacy	Evidence of demonstrated traditional use from published scientific literature or international monographs	Clinical studies

3. **Classifications:** In order for a HM to be assessed and evaluated under the appropriate registration pathway, HMs are proposed to be classified according to two key features or characteristics; the presentation of the product and the purpose for which it is administered:
  - ❖ Presentation: - If a claim to treat a major health condition is added, the product is classified under the HMR pathway. (Include list of conditions for which products are unlikely to get registered at the THR)

- For a product to be classified under the THR pathway only claims that are functional, structural, or therapeutic indications based on long-standing use are allowed. (Include examples of indications likely to be permitted in THR). Products that include claims of treating, diagnosing, preventing or curing of diseases are automatically classified under the HMR.

- Products under the THR pathway can only be presented as oral, external and inhalation preparation. Products under the HMR pathway may include any preparation type.

- ❖ Purpose: If a product requires the supervision of a medical practitioner, or a medical prescription, the product will be classified under the HMR pathway irrespective of the proposed indications.

## Abbreviations

CEO: Chief Executive Officer
DRA: Drug Regulatory Authority
DRRS: Drug and Registration Release Superintendent
EMA: European Medicines Agency
EMR: Eastern Mediterranean Region
GCC: Gulf Cooperation Council
HM: Herbal Medicine
HMR: Herbal Medicine Registration
HP: Health Product
IDI: International Development Ireland
IMB: Irish Medical Board
IRF: Information Request Form
ISO: International Organisation for Standardisation
KDFCA: Kuwait Drug and Food Control and Authority
MHRA: Medicines and Healthcare products Regulatory Agency
MOH: Ministry of Health
NHRA: National Health Regulatory Authority
PPC: Pharmaceutical Product Classification
SCH: Supreme Council of Health
SFDA: Saudi Food and Drug Authority
SOP: Standard Operating Procedure
SWOT: Strengths, Weaknesses, Opportunities and Threats
THR: Traditional Herbal Registration
TORC: Theory of Organisational Readiness for Change
U.S FDA: United States Food and Drug Administration
UK: United Kingdom
US: United States
WHO: World Health Organisation
