# Supplementary material for: A qualitative exploration of Bahrain and Kuwait herbal medicine registration systems: policy implementation and readiness to change
Source: J Pharm Policy Pract. 2019 Oct 9;12:32. doi: 10.1186/s40545-019-0189-7 (PMC6784343; doi:10.1186/s40545-019-0189-7)
Supplement: Supplementary file 1 — Description of data sources used in the illustration of findings in case 1 and case 2 (DOCX 14 kb) [file 40545_2019_189_MOESM1_ESM.docx]

**Additional file 1: Description of data sources used in the illustration of findings in case 1 and case 2**

**Table 1**

**Description of sources of data used in each case**

| Cases themes | | Sources of data used |
| --- | --- | --- |
| Case 1 | Context, Actors, Content and  Process in the development and  implementation of the  PPC policy in the NHRA | Data from interviews with 5 reviewers (KI1, KI2, KI3, KI4, KI5) and 3 managers (KI6,  KI7, KI8)  Document review of the Economic Vision 2030, Law (18) of 1997 With Respect  the Practice of Pharmacists and Pharmaceutical Centres, IDI Technical Support  Services for the Development, Operations and Management of the NHRA, Decree  (9) in relation to Classifying Pharmaceutical Products and Health Products, Health  Products Checklist, Medicines Checklist, Strategic Plan (2016 2020) and NHRA  annual report 2016, Pricing Guideline |
|  | The registration process  of HMs in the NHRA | Field notes from10 observations of 5 reviewers (KI1, KI2, KI3, KI4, KI5)  Document review of PPC guideline (2013), health  products registration requirements, medicines registration requirements and  Pricing Guideline |
|  | SWOT analysis for the HM  registration system in the  NHRA | Data from interviews with 5 reviewers (KI1, KI2, KI3, KI4, KI5) and 3 managers (KI6, KI7, KI8) |
| Case 2 | The registration process  of HMs in the KDFCA | Field notes from 19 observations of 9 reviewers (K19, KI10, KI11, KI12, KI13,KI14,  KI15, KI16, KI17)  Document review of Herbal Department Ministerial Decree (201/97), Dietary  Supplement Department Ministerial Decree (532/2002), Unclassified Department  Ministerial Decree (201/99) |
|  | Perceptions on the current  KDFCA’s HMs registration  system and readiness towards  implementation | Data from interviews with 9 reviewers (K19, KI10, KI11, KI12, KI13, KI14, KI15,  KI16, KI17) and 6 Managers (K118, K119, K120, K121, K122, K123) |

*HM* herbal medicine, *IDI* International Development Ireland, *KDFCA* Kuwait Drug and Food Control and Administration, *NHRA* National Health Regulatory Authority, *PPC* Pharmaceutical Product Classification, *SWOT* Strengths, Weaknesses Opportunities and Threats

Additional file 1: Description of data from documents, field notes and interviews used for the illustration of findings in case 1 and case 2
