# Supplementary material for: A qualitative exploration of Bahrain and Kuwait herbal medicine registration systems: policy implementation and readiness to change
Source: J Pharm Policy Pract. 2019 Oct 9;12:32. doi: 10.1186/s40545-019-0189-7 (PMC6784343; doi:10.1186/s40545-019-0189-7)
Supplement: Supplementary file 2 — An analysis of the Context, Actors, Content and Process in the development and implementation of the Pharmaceutical Product Classification policy in the Bahraini drug regulatory authority (DOCX 58 kb) [file 40545_2019_189_MOESM2_ESM.docx]

**Additional file 2: An analysis of the Context, Actors, Content and Process in the development and implementation of the Pharmaceutical Product Classification policy in the Bahraini drug regulatory authority**

**Figure 1**

**Chronological progress of the Pharmaceutical Product Classification policy at the Bahraini drug regulatory authority**

-Launch of the 2030 Economic Vision

-Suggestions to separate regulation of the entire healthcare system from the MOH

-Transition from the MOH to the NHRA was complete

- Employees facing difficulties with registering HMs according to old MOH regulations

- Finalisation of the PPC guideline

- Implementation of the guideline as a reference

- Agents resisting to comply with the guideline

-HMs registration at the MOH

- All HMs reviewed as medicines per Law (18) of 1997

- Classification law did not exist

-Establishment of the NHRA as an independent body responsible for the regulation of the entire healthcare system in Bahrain as per Law (38) of 2009

-Transition of regulatory procedures from MOH to NHRA

- Three-year contract with the IDI to improve health regulations in the NHRA

- Establishment of a committee to produce a classification guideline

ABSENCE OF A CLASSIFICATION

- Binding of the guideline (policy) as per Decree (9) of 2016

-Immediate implementation of the policy by reviewers and establishment of classification committee

- Agents were notified to legally comply with the policy

EXISTENCE OF THE PPC POLICY

*HMs* herbal medicines, *IDI* International Development Ireland, *MOH* Ministry of Health, *NHRA* National Health Regulatory Authority

Additional file 2: Data from the analysis of collected documents and interview transcripts on the chronological progress of the Pharmaceutical Product Classification policy in the Bahraini drug regulatory authority
