# Supplementary material for: A qualitative exploration of Bahrain and Kuwait herbal medicine registration systems: policy implementation and readiness to change
Source: J Pharm Policy Pract. 2019 Oct 9;12:32. doi: 10.1186/s40545-019-0189-7 (PMC6784343; doi:10.1186/s40545-019-0189-7)
Supplement: Supplementary file 3 — An analysis of the production stages of the Pharmaceutical Product Classification guideline at the Bahraini drug regulatory authority (DOCX 31 kb) [file 40545_2019_189_MOESM3_ESM.docx]

**Additional file 3: An analysis of the production stages of the Pharmaceutical Product Classification guideline at the Bahraini drug regulatory authority**

**Figure 1**

**Production stages of the Pharmaceutical Product Classification guideline at the Bahraini drug regulatory authority, with participants’ quotes**

*CEO* Chief Executive Officer, *DRAs* drug regulatory authorities, *GCC* Gulf Cooperation Council, *HMs* herbal medicines, *ISO* International Organisation for Standardisation, *MOH* Ministry of Health

Additional file 3: Data from the analysis of interview transcripts on the production stages of the Pharmaceutical Product Classification guideline at the Bahraini drug regulatory authority
