# Supplementary material for: A qualitative exploration of Bahrain and Kuwait herbal medicine registration systems: policy implementation and readiness to change
Source: J Pharm Policy Pract. 2019 Oct 9;12:32. doi: 10.1186/s40545-019-0189-7 (PMC6784343; doi:10.1186/s40545-019-0189-7)
Supplement: Supplementary file 4 — An analysis of facilitators and barriers in the development and implementation stages of the Pharmaceutical Product Classification policy in the Bahraini drug regulatory authority (DOCX 28 kb) [file 40545_2019_189_MOESM4_ESM.docx]

**Additional file 4: An analysis of facilitators and barriers in the development and implementation stages of the Pharmaceutical Product Classification policy in the Bahraini drug regulatory authority**

Table 1

Perceived facilitators in the development stage of the Pharmaceutical Product Classification policy at the Bahraini drug regulatory authority, with participants’ quotes

| Themes | Facilitators | Participants quotes |
| --- | --- | --- |
| Management and  collaboration | NHRA seeking to build good reputation as a newly  established entity  Coordination with external organisations  Teamwork among NHRA officials and external  experts | *“We learned from them [external experts] so much. They were very open in sharing their knowledge and the communication with them was easy. You know we had the chance to benefit from great minds so we tried to benefit from this as much as we can” (KI7)* |
| Leadership | Key figures planning and guiding the development  process and providing officials with support and  encouragement | *“We had continuous support from the CEO she understands us and she supported us” (KI5)* |
| Resources | Availability of funding  Availability of skilled external experts  Availability of data and informative DRAs websites | *“To be realistic, financial support it was the most important part, because there would be no contract with the Irish group without money, and without the experts it would be difficult to make the guideline” (KI1)* |
| Nature and content  of the policy | Not mentioned |  |
| Political and social  influences | Incoherent existing policy causing controversy and  existing of international classification policies in  other countries providing justification for the  decision to issue a classification  Bahrain being a member of the GCC facilitating  communications and inquiries with other members  NHRA independence and freedom from the  political and commercial influence of the MOH  SCH support in approving the policy efficiently | *“Being part of the GCC and during our communications with the GCC countries for central registration, we learned that certain guidelines must exist which will make our life easier, and availability of a classification system in Saudi Arabia helped a lot “(KI8)*  *“We are independent from the MOH, so the production of new policies and guidelines doesn’t require the MOH approval and the long process of approving polices. Internally at least we can produce our own policies and guidelines and the MOH can’t interfere with this” (KI4)*  *“We are not alone, we have the support of the Supreme Council, and they strengthened our role as an independent organisation and they enabled all our regulations to come into action very quickly. We felt that they had our back” (KI7)* |
| Staff morals and  performance | Commitment of committee members in finalising  the production of the guideline on time | “*To be honest, we were efficient; we worked very hard to accomplish the guideline. We had consistent meetings, sessions after sessions, with dedication and team-work effort it was possible to deliver the guideline on the right time” (KI7)* |

*DRAs* drug regulatory authorities, *CEO* Chief Executive Officer, *GCC* Gulf Cooperation Council, *MOH* Ministry of Health, *NHRA* National Health Regulatory Authority, *SCH* Supreme Council of Health

Table 2

Perceived barriers in the development stage of the Pharmaceutical Product Classification policy at the Bahraini drug regulatory authority, with participants’ quotes

| Themes | Barriers | Participants quotes |
| --- | --- | --- |
| Management and collaboration | Not mentioned |  |
| Leadership | Not mentioned |  |
| Resources | Lack of sufficiently trained and  experienced NHRA staff | *“Our experience in Chemistry and conventional medicines is more than herbals. Establishing classification or even policies to regulate herbs is extremely difficult; the topic is not just black and white, it is a complicated subject and we don’t have the expertise. Unfortunately, we didn’t receive training on decision-making techniques. It all depended on our personal effort and reading and asking other experts” (KI3)* |
| Nature and content of the policy | Diversity of worldwide herbal  regulations and lack of a  universal classification for  HMs | *“The problem with herbs that, for example USA licenses it as food supplement, UK however classify it differently, we import our product from both countries, how could we adopt a reasonable classification?” (KI1)* |
| Political and social influences | Not mentioned |  |
| Staff morals and performance | Not mentioned |  |

*HMs* herbal medicines, *NHRA* National Health Regulatory Authority, *UK* United Kingdom, *USA* United States of America

Table 3

Perceived facilitators in the implementation stage of the Pharmaceutical Product Classification policy at the Bahraini drug regulatory authority, with participants’ quotes

| Themes | Facilitators | Participants quotes |
| --- | --- | --- |
| Management and  collaboration | Teamwork and cooperation  between officials  Setting an adaptation period  for pharmaceutical companies  to comply with the new  system | *“We see each other more than we see our family; we spend more hours during the day with each other than our families, so we respect each other very much this is important we also helped each other, if I was sick, my colleague would cover my duties until I come back. I did the same. When you work in a workplace like this, it makes it easier to implement procedures effectively” (KI2)*  *“We as a team used to meet once every two weeks to see how the implementation is going and whether any difficulties has occurred while implementing it through receiving the files and classifying the product… We keep records of cases we can’t classify with the guideline so we don’t forget when we update the guideline” (KI4)*  *“We gave them [agents] some time to provide us with the requirements for their registered products according to the new guideline. We understood it is new for them, we gave them some time to understand and cope” (KI3)* |
| Leadership | Effective internal leadership  and support from key figures | *“The role of the CEO normally is to stand at the top of the pyramid and the rest takes order, our CEO is different, she helped us with difficult registration issues, even with the guideline sometimes we had to make different decisions”(KI2)* |
| Resources | Availability of international  references and availability of  informative international DRAs’  websites  No training was required on how to  implement the policy | *“I was searching a lot online because when you search you find everything you need. Overall if we don’t have a reference for some product or information is not in the guideline, or we are unsure, we always check our referenced trusted authorities’ websites” (KI1)*  *“Already the reviewers that needs to implement the guideline in practice were involved in the production process, so they are aware of the guideline and understands how to implement it effectively. They [old reviewers] taught the new reviewers everything, how to use the guideline and why it is important that they use it” (KI7)* |
| Nature and content of the policy | Availability of a clear guideline  The guideline being based on  classifications of countries that  Bahrain imports from | *“It is really great to have a guideline it makes my life easier. You can’t always depend on other countries’ websites, sometimes you need your own guideline. A lot of information inside the guideline is from countries we import from, so it is easier when we request for documents. The guideline is here to help us as regulators and help the agents as well. For agents, you can’t just submit something and just wait it might be accepted or rejected, there are specifications, knowing where the product can be classified from the beginning saves a lot of time and effort” (KI1)* |
| Political and social influences | Inability of the MOH to influence  decisions made by the NHRA  Binding the guideline and forcing  sanctions on violators | *“The separation from the MOH increases the integrity of the drug regulatory and gives protection from interference of special interests… We also enforced penalties on anyone who try to break up this regulation or other regulations as well, so people are really careful to comply” (KI8)* |
| Staff morals and performance | Staff motivation and devotion to  effectively implement policies in  order to protect the public | *“You need excellent reviewers, but you also need reviewers who are dedicated to make the implementation work” (KI8)* |

*CEO* Chief Executive Officer, *MOH* Ministry of Health, *NHRA* National Health Regulatory Authority

Table 4

Perceived barriers in the implementation stage of the Pharmaceutical Product Classification policy at the Bahraini drug regulatory authority, with participants’ quotes

| Themes | Barriers | Participants quotes |
| --- | --- | --- |
| Management and  collaboration | lack in providing a clear plan for  policy implementation and  allocation of resources | *“We barely had the time to finalise the guideline, we needed it urgently, we thought that as soon as we have it we will use it and it will solve many problems, and it did solve many problems, but yes we should’ve had a better plan to make the implementation work even better”(KI6)* |
| Leadership | Not mentioned |  |
| Resources | Lack of expertise in HMs | *“Herbs are the most difficult products that we have. We need expertise, which we do lack right now. That’s why we depend on external regulations” (KI8)* |
| Nature and content  of the policy | Diversity in HMs classifications  worldwide and the continuous  change in HMs regulations  No use of scientific evidence in the  development of the policy | *“HMs regulations keep changing. Of course it needs improvements, but even with this second version guideline, I think that it still needs improvements. But I can’t make any further improvements because the problem is that every country has its own method of assessment and their own classification system, with the same herb you find it banned in one country, but another country it is classified as health product, even the type of classification is different. For example some country has what is called “traditional herbal medicine” other countries don’t” (KI6)*  *“The Irish reference states that a product cannot have more than five herbs in it. If it does then it will not get registered as HP, but as a medicine. We searched this information, we couldn’t find that reference other than Saudi Arabia who had it as well, but based on what, this we couldn’t know. So we are changing this rule now” (KI7)* |
| Political and social  influences | Resistance from agents to comply  with the new system | *“It is taking them [agents] a long time to adapt; we still face issues with agents not aware of the current regulations. But we are getting there, change needs time” (KI4)* |
| Staff morals and  performance | Not mentioned |  |

*HMs* herbal medicines, *HP* health product

Additional file 4: Data from the analysis of interview transcripts on perceived facilitators of, and barriers to the development and implementation of the Pharmaceutical Product Classification policy in the Bahraini drug regulatory authority
