# Supplementary material for: A qualitative exploration of Bahrain and Kuwait herbal medicine registration systems: policy implementation and readiness to change
Source: J Pharm Policy Pract. 2019 Oct 9;12:32. doi: 10.1186/s40545-019-0189-7 (PMC6784343; doi:10.1186/s40545-019-0189-7)
Supplement: Supplementary file 5 — An analysis of the classification and registration process of herbal products and herbal medicines at the Bahraini drug regulatory authority, including similarities and differences between the two pathways (DOCX 21 kb) [file 40545_2019_189_MOESM5_ESM.docx]

**Additional file 5: An analysis of the classification and registration process of herbal products and herbal medicines at the Bahraini drug regulatory authority, including similarities and differences between the two pathways**

**(A) Classification**

The agent can request for a classification appointment using the online Pharmaceutical Product Classification (PPC) application at the National Health Regulatory Authority’s (NHRA) website. The agent is given an exact time and date to submit materials consisting of signed and stamped cover letter and application form, artwork of the product’s outer-packaging and inner label, copy of the composition certificate issued from the manufacturing company, one product sample and a payment of 50 USD. On the day of the appointment, the reviewer validates submitted materials and prepares a report stating an initial classification decision whether the product should be classified as a herbal medicine or a herbal product upon interpreting the totality of four factors based on the PPC policy.

1) If the product explicitly claims to treat or prevent a disease, it will be considered as a herbal medicine and any claims made for herbal products should be consistent with available evidence regarding the safety and traditional use of those products e.g. published peer-reviewed scientific literature. 2) Herbal products cannot be sterile, and/or administered by injection and products with these characteristics will be considered as herbal medicines. 3) If the product is subject to a medical prescription or/and requires the intervention of a healthcare practitioner, the product will be considered as a herbal medicine. 4) The classification guideline contains a list of herbal substances, which are considered not suitable as herbal products but may be considered as herbal medicines.

The classification committee consisting of the reviewer responsible for the product’s classification, an external specialist (depending on the nature of the product) and a senior pharmacist makes the final classification decision. The decision is made upon reviewing the reviewer’s report ensuring that the product is classified according to the most suitable classification based on the four classification factors. Overall, it takes approximately one month for the product to be classified in the NHRA. Once the product is classified, it must be submitted for registration in the related department.

**(B) Submission**

Similar to classification, for herbal medicine or herbal product registration, the agent must request an appointment through the NHRA official website to submit administrative, quality, safety and efficacy requirements documents to the relevant department.

**Table 1**

**Similarities and differences in the registration requirements of herbal products and herbal medicines extracted from Law (18) of 1997 With Respect the Practice of Pharmacists and Pharmaceutical Centres**

| **Regulatory requirements** | **Herbal product** | **Herbal medicine** |
| --- | --- | --- |
| **Administrative** | | |
| Legalised Good Manufacturing Practice Certificate | **✓** | **✓** |
| Finished product sample and leaflet | **✓** | **✓** |
| Original Legalised Free-Sale Certificate or Certificate of Pharmaceutical Product | **✓** | **✓** |
| Original legalised Price Certificate | **X** | **✓** |
| **Quality** | | |
| Certificate issued by the company stating that the product does not contain or contains the allowed percentage of heavy metals | **✓** | **✓** |
| Certificate of Suitability for the active substance | **X** | **✓** |
| Raw materials specifications | **X** | **✓** |
| Finished product specifications | **✓** | **✓** |
| Composition of the product | **✓** | **✓** |
| Certificate of Analysis | **X** | **✓** |
| Stability study | **X** | **✓** |
| Declaration of alcohol and deceleration of pork free content | **✓** | **✓** |
| **Safety and efficacy** | | |
| Clinical studies/ scientific references | **✓**  (scientific references) | **✓**  (clinical studies) |

Requirements for herbal products are less demanding than of herbal medicines (Table 1). For example, unlike the herbal medicine, a herbal product does not require clinical studies to demonstrate its’ safety and efficacy, the submission of published scientific references stating the traditional use is sufficient.

On the appointment day, the reviewer validates the submitted documents and divides it into two sections, the safety and efficacy section which is queued for reviewing by the scientific reviewer and the quality section which is queued for assessing in the laboratory.

**(C) Evaluation**

Two evaluation routes for herbal medicines and herbal products registration exists in the NHRA; the verification and the abridged evaluation.

The Verification evaluation route is used to reduce duplication of effort by agreeing that Bahrain will allow herbal medicines and herbal products to be locally marketed once they have been authorised by two or more recognised competent regulatory authorities in countries such as Australia, Canada, United Kingdom, and some countries in Western Europe. In this route, the reviewer only ‘verifies’ that the product intended for registration has been duly registered as declared in the application and that the product characteristics (formulation composition) and the presenting information (use, dosage, precaution) for local marketing conforms to that agreed in the reference authorities.

The Abridged evaluation route applies to herbal medicines and herbal products that do not fall under the verification route. However, it conserves resources by not re-assessing all scientific supporting data that have been reviewed and accepted elsewhere, but includes an ‘abridged’ independent review of the product in terms of its use under local conditions, this might include review of the climatic conditions, benefit risk assessment in relation to use in local ethnic population, cultural/medical practice and patterns of disease and nutrition. It is a requirement that the product is registered for a minimum of twelve months in the country of origin and approval by a recognised regulatory authority elsewhere is pre- requisite before the local authorisation can be granted. In both routes, an assessment template is completed by the appointed reviewer.

In both pathways, products samples are sent to the laboratory for chemical and physical analysis as per their finished product’s specifications. Most agents request that samples are analysed in a private laboratory (analysis takes fifteen days) which is accredited by the NHRA to avoid long waiting periods that might take up to three months in the governmental laboratory. No interaction is allowed between the agent and the laboratory and once results are completed and open for review, they are sent directly to the responsible reviewer.

Once the scientific and laboratory assessment of the product is complete, queries or concerns relevant to the product’s quality, safety and efficacy are generated. The queries are sent to the agent by the reviewer electronically through completing an Information Request Form (IRF).

**(D) Authorisation**

Once herbal medicines have been assessed and tested, the product is transferred to the pricing department and a certain price within the required limits must be assigned according to the Pricing Guideline’s formula; if the herbal medicine costs less than 50 USD, the profit margin is 35%, and if the medicine costs more than 50 USD, the profit margin is 20%. Herbal products are not priced, and the agent can freely price the product without any restrictions.

The final decision to approve a herbal medicine or a herbal product is carried out by the licensing committee, which consist of a senior pharmacist, the reviewer who reviewed the file, the Chief of the Pharmaceutical Product Regulation Department and an external reviewer. An assessment template completed by the appointed reviewer during the assessment stage is presented to the committee to provide a standardised content and format of the data and shows the reviewer’s recommended decision. The committee evaluates the report and the final decision is made collectively. An approval certificate is then issued and signed by the Chief of the Pharmaceutical Product Regulation Department.

Upon approval of the committee, the agent can import the product’s shipment from the country of origin. The NHRA follows the Invoice Clearance Procedure Guideline to release herbal products and herbal medicines into the market.

Additional file 5: Data from the analysis of fieldnotes and documents on the classification and registration process of herbal products and herbal medicines at the Bahraini drug regulatory authority
