# Supplementary material for: A qualitative exploration of Bahrain and Kuwait herbal medicine registration systems: policy implementation and readiness to change
Source: J Pharm Policy Pract. 2019 Oct 9;12:32. doi: 10.1186/s40545-019-0189-7 (PMC6784343; doi:10.1186/s40545-019-0189-7)
Supplement: Supplementary file 6 — An analysis of Strengths, Weaknesses, Opportunities and Threats of the current HMs registration system at the Bahraini drug regulatory authority (DOCX 19 kb) [file 40545_2019_189_MOESM6_ESM.docx]

**Additional file 6: An analysis of Strengths, Weaknesses, Opportunities and Threats of the current HMs registration system at the Bahraini drug regulatory authority**

**Table 1**

**Perceived Strengths, Weaknesses, Opportunities and Threats in the current HMs registration system at the Bahraini drug regulatory authority, with participants’ quotes**

| Strengths (internal conditions) | Participants quotes |
| --- | --- |
| Motivation of the regulatory authority to  improve making it a trustworthy authority | *“We are aiming to be number one regulatory authority in the Gulf, so we are always trying*  *to improve our procedures, our system, going over old policies and advance them” (KI3)* |
| Transparency and honesty of the review  procedure | *“We make sure that we are transparent as much as possible, this is very important to gain*  *public trust” (KI8)* |
| Existence of committee for scientific  assessment | *“The final decision for approving medicines are made by the scientific committee, this has*  *the advantage of ensuring the availability of experienced staff and discuss issues that may*  *not be tackled by only one reviewer” (KI1)* |
| Availability of guidelines and SOPs | *“We have guidelines, we follow our own SOPs, we have registration requirements that are*  *clear, these helps a lot, they help us carryout the registration procedure consistently*  *“(KI8)* |
| Availability of an electronic system | *“Having an electronic system to register and track applications, to record decisions of*  *applications and you, this provide better handling for information and saves time” (KI6)* |
| Weaknesses (internal conditions) | **Participants quotes** |
| Lack in the organisational structure and  hierarchy | *“I think that the organisational structure could be improved to give a higher authority to*  *certain positions. For example, the structure needs to be more hierarchal, there is still*  *some gaps in the structure of the different departments of the Pharmaceutical Product. I*  *think it is better to have one manager for each department, like for HPs, medicines,*  *renewals and so on, like this I think it is easier for reviewers to reach their managers*  *instead of having one manager for all departments” (KI2)* |
| Lack of training in HMs regulations | *“We do have financial resources but it is very restricted and for all employees under all*  *departments in the NHRA not specifically for Pharmaceutical Product department. And*  *what happens is that the [names another department in the NHRA] takes the most of the*  *training being a newly established department that needs improvements. How can I*  *improve the authority without continuous training and continuous knowledge about what is*  *happening in the regulatory aspects worldwide?” (KI3)* |
| Extreme lack of human resources | *“The extreme limitation in staff is becoming a serious problem, it’s causing us a hard time*  *finishing deadlines on time, because you have so much work to do, the type of work we do*  *needs time, you can’t rush things. We need more staff, that’s for sure” (KI4)* |
| Opportunities (external conditions) | **Participants quotes** |
| Independent authority | *“We are independent from the MOH, so the production of new policies and guidelines*  *doesn’t require the MOH approval and the long process of approving policies. Internally*  *at least we can produce our own policies and guidelines and the MOH can’t intervene with*  *this” (KI8)* |
| Working in collaboration with regional and  international agencies | *“Cooperation with international authorities is very important, we have several*  *collaborations with agencies for example the WHO, which also have many competent*  *followers, and we are growing along with the global advancements of these followers”*  *(KI2)* |
| Expand through GCC cooperative efforts | *“Being part of the GCC and during our communications with the GCC countries for central*  *registration, we learn that certain guidelines must exist which will make our life easier”*  *(KI6)* |
| Threats (external conditions) | **Participants quotes** |
| Poor funding resulting in lack of significant  regulatory procedures | *“We need experts on pharmacovigilance, more staff and connections with referenced*  *countries and hotlines, and more importantly, we need a full team and a guideline to start*  *with, this all needs more money” (KI1)* |
| Open market | *“When consumers do not find what they need from the market they would easily order HMs*  *online and who knew what these products include they might’ve contained banned and*  *dangerous substances, these are not tested” (KI5)* |
| Increased number of substandard and  counterfeit HMs | *“Unsafe HMs are incredibly increasing all over the world, many products we received did*  *not meet the required quality or safety, we need to be very careful with herbs and very*  *thorough in our review” (KI4)* |

*GCC* Gulf Cooperation Council, *HMs* herbal medicines, *HPs* health products, *MOH* Ministry of Health, *SOPs* Standard Operating Procedures, *WHO* World Health Organisation

Additional file 6: Data from the analysis of interview transcripts on Strengths, Weaknesses, Opportunities and Threats of the current HMs registration system at the Bahraini drug regulatory authority
