# Supplementary material for: A qualitative exploration of Bahrain and Kuwait herbal medicine registration systems: policy implementation and readiness to change
Source: J Pharm Policy Pract. 2019 Oct 9;12:32. doi: 10.1186/s40545-019-0189-7 (PMC6784343; doi:10.1186/s40545-019-0189-7)
Supplement: Supplementary file 7 — An analysis of the registration process of HMs at the Herbal Department, Dietary Supplement Department and Unclassified Department in the Kuwaiti drug regulatory authority, including similarities and differences between the three departments (DOCX 19 kb) [file 40545_2019_189_MOESM7_ESM.docx]

**Additional file 7: An analysis of the registration process of HMs at the Herbal Department, Dietary Supplement Department and Unclassified Department in the Kuwaiti drug regulatory authority, including similarities and differences between the three departments**

**(A) Submission**

The Kuwait Drug and Food Control and Administration (KDFCA) does not have a system for appointments, product’s dossier is submitted directly to the Director of the KDFCA with an official request letter from the local agent to register the product in the specified department, where the Director officially accepts the dossier and transfers it to the Drug and Registration Release Superintendent (DRRS) who transfers the dossier to the requested Department. The validation of the dossier begins in the same day as receiving whereby the reviewer verifies submitted administrative, quality, safety and efficacy requirements. Requirements for HMs in the Dietary Supplement and Unclassified Departments are less demanding than requirements in the Herbal Department (Table 1).

**Table 1**

**Summary comparison of key registration requirements of HMs in each department extracted from Herbal Department Ministerial Decree (201/97), Dietary Supplement Department Ministerial Decree (532/2002) and Unclassified Department Ministerial Decree (201/99)**

| **Regulatory requirements** | **Herbal Department** | **Dietary Supplement Department** | **Unclassified Department** |
| --- | --- | --- | --- |
| **Administrative** | | | |
| Application form completed by the agent | **✓** | **X** | **X** |
| Checklist completed and signed by the manufacturer | **✓** | **X** | **X** |
| Finished product sample and leaflet | **✓** | **✓** | **✓** |
| Original Legalised Free-Sale Certificate or Certificate of Pharmaceutical Product | **✓** | **✓** | **✓** |
| Status of registration of the product in the country of origin | **✓** | **✓** | **✓** |
| List of countries where the product is registered with registration dates and numbers | **✓** | **✓** | **✓** |
| Original legalised price certificate | **✓** | **✓** | **X** |
| **Quality** | | | |
| A “Free From” certificate issued by the manufacturer | **✓** | **X** | **X** |
| Certificate issued by the company stating that the product does not contain or contains the allowed percentage of heavy metals | **✓** | **X** | **X** |
| Finished product specifications | **✓** | **X** | **X** |
| Certificate of analysis of finished product | **✓** | **✓** | **✓** |
| Complete stability study on three production batches | **✓** | **X** | **X** |
| Raw materials (both active substances and excipients) specifications and certificate of analysis and certificate of suitability of the active ingredients | **✓** | **X** | **X** |
| Original legalised pork free/ alcohol free certificate | **✓** | **✓** | **✓** |
| **Safety** | | | |
| Safety studies from approved international authority or scientific references | **✓** | **X** | **✓** |
| **Efficacy** | | | |
| Clinical studies/ scientific references | **✓** | **X** | **X** |

**(B) Evaluation**

In all three departments, scientific assessment and laboratory analysis both proceed in parallel. For the scientific assessment, reviewers start the assessment process by verifying the submitted documents and making sure that they are in a clear and unambiguous order within the file. In all three departments reviewers perform an ‘abridged’ review for assessing the documents submitted concentrating on assessing submitted quality data in relation to climatic conditions, finished product specifications and Certificate of Analysis to ensure that the quality data submitted are within the specifications. Other data are not investigated unless a query is raised on a specific product. An assessment template is used by reviewers in each department to provide a standardised content and format of the data assessed to present a scientific study report.

Not all HMs in the three departments undergoes the analysis because the authority relies on the Certificate of Analysis submitted by the agent from the manufacturer. However, all sexual and slimming herbal products must pass the testing to check for adulteration of products with other substances such as Sebutramine or Sildenafil. After the analysis is complete, the analysists prepare a quality assessment report stating whether the product has passed or failed the analysis. The report is then transferred to the concerned registration department where the reviewer completes the scientific study report.

Questions are collected as they arise during the scientific assessment and laboratory testing and are provided to the agent. Unlike the Herbal Department, the Unclassified and Dietary Supplement Departments does not place any limits on the agent’s response time. In all three departments, once the response from the company is received, it is reviewed by the same appointed reviewer who initially reviewed the registration dossier, and accordingly a final decision is taken.

**(C) Authorisation**

In all three departments the scientific reviewer proposes the final approval decision individually which is signed off by the Head of each department and the DRRS.

Products registered at the Unclassified Department do not enter the pricing stage and are priced according to the company’s desires. For products in the other two departments a pricing committee consisting of the Head of the Pricing Department and at least two reviewers decide on the price which is usually the export price to the Saudi market.

Once the product is approved (for HMs in the Unclassified) and priced (for HMs in the Herbal and Dietary Supplement Departments) the local agent is provided a first release to order the first shipment of their registered product. For HMs in the Dietary Supplement and Unclassified Departments, the agent submits the shipment invoice to the reviewer to grant the second release and the products can be released into the market. For HMs in the Herbal Department, in addition to the submission of the shipment invoice, the agent is required to submit a sample from the shipment to the laboratory for analysis to check for any heavy metals and microbial contamination. The test results are sent back to the same appointed reviewer who provides the second release and the product can then be released into the market.

Additional file 7: Data from the analysis of fieldnotes and documents on the registration process of HMs in the Herbal Department, Unclassified Department and Dietary Supplement Department at the Kuwaiti drug regulatory authority
