# Supplementary material for: A qualitative exploration of Bahrain and Kuwait herbal medicine registration systems: policy implementation and readiness to change
Source: J Pharm Policy Pract. 2019 Oct 9;12:32. doi: 10.1186/s40545-019-0189-7 (PMC6784343; doi:10.1186/s40545-019-0189-7)
Supplement: Supplementary file 8 — An analysis of factors affecting the Kuwaiti drug regulatory authority’s readiness to implement the proposed recommendations (DOCX 21 kb) [file 40545_2019_189_MOESM8_ESM.docx]

**Additional file 8: An analysis of factors affecting the Kuwaiti drug regulatory authority’s readiness to implement the proposed recommendations**

**Table 1 Contextual factors affecting the Kuwaiti drug regulatory authority’s readiness to implement the proposed recommendations, with participants’ quotes**

| Contextual factors | | Participants quotes |
| --- | --- | --- |
| Policies and procedures | Reviewers suggesting that  current management must  be changed | *“The authority needs young minds, people who are motivated to improve the place. Giving someone a high position comes with responsibilities and improving the system is one of them. When this person has reached a very advanced age, they do the minimum, and do not really care whether this place needs new regulations or guidelines, they are not bothered to make progress, and the place goes back in time and becomes underdeveloped. Renewing the blood, improves systems” (KI10)* |
|  | Increase motivation in  improving the system  through rewards and  promotions | *“It is the nature of our work you know, we do not receive rewards and bonuses when we succeed in improving the system or when we work really hard, we get promoted because of the years we serve, so the staff is not motivated to improve the system, they will gain nothing from that”(KI11)* |
|  | Increase the interaction  between management and  reviewers | *“They [management] need to know the issues and be ready to hear the proposed solutions. Management are not working on their own, we are under them and we carry most of the critical procedures, we actually do all the work, if we don’t share what views we have with them, how would they know? At the end, we all work together here, at the same place, and the reputation of this place touches us all. It is the managements’ benefit to support us” (KI11)* |
|  | Management willingness to  involve whoever is  necessary | *“There should be direct involvement between us [managers] and reviewers in policymaking and implementation, because I believe that this can contribute positively on the policy and we [managers] can gain from their [reviewers] experience. There is no denial in this. We are willing to involve any employee we could benefit from” (KI14)* |
|  | Approving the policy as  a Ministerial Decree | *“High ranking approval. I can prepare a guideline, train people, have it ready, but if you don’t have the support of the high ranking, and get it approved, either as an administrative or they send it to the Ministry, to get it approved as a Ministerial Decree, basically you’re standing still” (KI20)* |
|  | Separate the KDFCA from  the MOH | *“The best thing about it [KDFCA independency from MOH] will be how quick to introduce and implement any new legislation. And it’s the easiness of improving and updating, without the formalities of the Ministry. And it gives an extra strength to the power of the administration to have overall control over anything related to medicine. This is the trend worldwide to have the medicine agency separate from the Ministry” (KI20)* |
| Past experience | The policy should be  compatible with other  countries | *“We do not want to have our own guidelines that cannot be implemented because it is very different than the guidelines of the countries we are importing from you know” (KI11)* |
|  | Must take into account the  exporting countries’  classifications when  preparing the policy | *“…the documents that the agent will be able to provide depends on how the country [exported country] classify this product. The guideline that you will be implementing, it will cause problems if it is not aligned with international classifications and will be hard to implement in practice. If you think about the main problem, it is the differences in classifications that the different countries around the world have. This cause the problems and companies take advantage of this” (KI23)* |
|  | Continuous update of the  policy and discussions  with employees to discuss  any issues | *“We must not make the same mistake as with ministerial decrees that are now very old because they were not updated. With the guideline, we will have to review the guideline and check for updates in other international guidelines and see if our guideline would need reviewing as well” (KI10)* |
|  | Providing an adaptation  period for agents | *“We can give them [agents] like a period of time to adapt themselves to the new routine” (KI9)* |
| Organisational resources | Increase staff and employ  specialists | *“We will need more crew. Re-classifications mean new registrations. You are asking the agent to provide with all required documents like a new file and will need to register it all over again according to our (herbal unit) registration requirements” (KI9)*  *“Not every pharmacist is familiar with all the herbs so the specialist is very important especially in the terms used for herbs and medicinal plants. You will have guidelines, so that is fine, but when someone argues on why you are implementing this thing, the specialist will need to explain it to the agents. That is why it is important to have specialists who truly understand why the guidelines are being implemented” (KI23)* |
|  | Apply training | *“You have updates every now and then, almost in the year you have more than one update, every reviewer is entitled to know about these updates, the changes that are happening, and why they’re happening, and how to implement them, because they are happening and we’re not isolated from the world. We need to be in line with international guidelines” (KI20)* |
|  | Sufficient financial  resources | *“The Ministry of Health holds an enormous amount of budget and it doesn’t have strategic plans and a specific system for budgeting, it finance all departments under it freely, financial resources won’t be a problem here” (KI21)* |
| Organisational culture | To limit mediation and  favouritism more than one  reviewer decides the  classification or  establishing a separate unit  for classification | *“See, it is very hard to have a professional relation with people you are already friends with. It is just the way it is. Call it social connection or cultural bond… That’s why I said it needs to be away from the agents…Mediation and making favours, can never work on a group, it works individually secretively between two people, so what we can do is that more than one reviewer decides the classification of the product not only one. To double-check it. If it is a group, then it will be managed correctly” (KI12)* |
|  | A separate office or a  reception that welcomes  the agents | *“we don’t have a private room when agent give us files, and discuss issues, everything is discussed here in front of other reviewers and other agents, and other people here can’t concentrate with the work they are doing” (KI14)* |
| Organisational structure | No change in the structure  if only a policy is  implemented | *“I don’t think that the structure will be affected at all. The guideline should be used by all departments, and whenever a HM is detected in the Dietary Supplement or Unclassified Departments, the reviewer must transfer it to the Herbal Department to get classified and registered properly” (KI13)* |
|  | Change in the structure if a  Classification Department  is implemented | *“…we are receiving many products that are difficult to classify so I think it will be necessary in the future that we establish a Classification Department to deal only with the classification of products” (KI23)* |

*HMs* herbal medicines, *KDFCA* Kuwait Drug and Food Control and Administration, *MOH* Ministry of Health

Additional file 8: Data from the analysis of interview transcripts on the five contextual factors affecting the Kuwaiti drug regulatory authority’s readiness to implement the proposed recommendations
